# Multimodal learning of noncoding variant effects using genome sequence and chromatin structure

**DOI:** 10.1093/bioinformatics/btad541

**Published:** 2023-09-05

**Authors:** Wuwei Tan, Yang Shen

**Affiliations:** Department of Electrical and Computer Engineering, Texas A&M University, College Station, TX 77843, United States; Department of Electrical and Computer Engineering, Texas A&M University, College Station, TX 77843, United States; Department of Computer Science and Engineering, Texas A&M University, College Station, TX 77843, United States; Institute of Biosciences and Technology and Department of Translational Medical Sciences, College of Medicine, Texas A&M University, Houston, TX 77030, United States

## Abstract

**Motivation:**

A growing amount of noncoding genetic variants, including single-nucleotide polymorphisms, are found to be associated with complex human traits and diseases. Their mechanistic interpretation is relatively limited and can use the help from computational prediction of their effects on epigenetic profiles. However, current models often focus on local, 1D genome sequence determinants and disregard global, 3D chromatin structure that critically affects epigenetic events.

**Results:**

We find that noncoding variants of unexpected high similarity in epigenetic profiles, with regards to their relatively low similarity in local sequences, can be largely attributed to their proximity in chromatin structure. Accordingly, we have developed a multimodal deep learning scheme that incorporates both data of 1D genome sequence and 3D chromatin structure for predicting noncoding variant effects. Specifically, we have integrated convolutional and recurrent neural networks for sequence embedding and graph neural networks for structure embedding despite the resolution gap between the two types of data, while utilizing recent DNA language models. Numerical results show that our models outperform competing sequence-only models in predicting epigenetic profiles and their use of long-range interactions complement sequence-only models in extracting regulatory motifs. They prove to be excellent predictors for noncoding variant effects in gene expression and pathogenicity, whether in unsupervised “zero-shot” learning or supervised “few-shot” learning.

**Availability and implementation:**

Codes and data can be accessed at https://github.com/Shen-Lab/ncVarPred-1D3D and https://zenodo.org/record/7975777.

## 1 Introduction

Genetic variation in the noncoding regions (estimated to constitute 98%–99% of human genomes) can be linked to traits and diseases (van Ouwerkerk *et al.* 2019, Frydas *et al.* 2022). Growing data and analyses from genome-wide association studies (GWAS) help identify and interpret such links (Zhang and Lupski 2015), along with evolving computational methods. GWAVA (Ritchie *et al.* 2014) prioritized noncoding variants by machine learning from various functional annotations (largely by the ENCODE Project Consortium). FunSeq2 (Fu *et al.* 2014) did so for cancer somatic variants using a weighted scoring scheme. Then epigenetic impact prediction was introduced for mechanistic interpretations, whereas deep learning models emerged. To predict DNA accessibility (specifically, DNase I hypersensitive sites), Basset (Kelley *et al.* 2016) applied convolutional neural networks (CNN) to learn from the data annotated by ENCODE (The ENCODE Project Consortium 2012) and Roadmap Epigenomics (Roadmap Epigenomics Consortium *et al.* 2015). To predict transcription factor (TF) binding sites and histone marks besides DNase I hypersensitive sites, DeepSEA (Zhou and Troyanskaya 2015) also used CNN and was later upgraded to Expecto (Zhou *et al.* 2018) and, recently, Sei (Chen *et al.* 2022) with more epigenetics data, more advanced models, and better performances. Focused on TF binding, DanQ (Quang and Xie 2016) combined CNN and recurrent neural networks (RNN; specifically, a bidirectional long short-term memory or bidirectional LSTM) to capture the long term dependencies between motifs, and TBiNet (Park *et al.* 2020) further included an attention mechanism for model interpretability. Recently a transformer, using a sparse self-attention mechanism for long sequences, was also applied to the topic (Zaheer *et al.* 2020).

As surveyed above, current machine learning methods for epigenetic impacts of noncoding variants only treat DNA as 1D sequences (often local ones up to 1000 base pairs or 1K bps). Although correctly focused on sequence determinants (Koohy *et al.* 2013, Boeva 2016, Ngo *et al.* 2019), they disregard that DNA sequences are packed in 3D chromosome structure whereas such 3D global perspective of DNA sequences is important to regulatory mechanisms and functional annotation of noncoding variants (Klemm *et al.* 2019, D’haene and Vergult 2021). Technologies such as Hi-C (Lieberman-Aiden *et al.* 2009) and ChIA-PET (Fullwood *et al.* 2009) reveal chromatin interactions such as long-range intra- and inter-chromosome enhancer–promoter interactions (Zhang *et al.* 2013, Dai *et al.* 2018, Chen *et al.* 2020) controlling gene expression. The relationship between epigenetic profiles and chromatin structures has also been characterized computationally for predicting chromatin 3D structures. (Qi and Zhang 2019, Dodero-Rojas *et al.* 2023, Tan *et al.* 2023).

In this study, we fill the gap by incorporating both 1D local sequences and 3D global structures (across all chromosomes) into predicting epigenetic impacts of noncoding variants. The approach of multimodal data integration is currently lacking for the topic, although some recent methods predicted how noncoding variant sequences can change chromatin conformations (Trieu *et al.* 2020, Zhou 2022) and GraphReg predicted gene expression using interactions within 2 megabases (Karbalayghareh *et al.* 2022). Specifically, we first statistically test the hypothesis underlying the approach and verify that disparity between the levels of sequence (feature) similarity and epigenetic profile (label) similarity can be attributed to chromatin interactions. Given the rationale verified we then introduce a series of models combining the embedding of 1D local sequences (through CNN, RNN, and transformer) and the embedding of 3D global structures/interaction intensity graphs [through various graph neural networks (GNNs)], while overcoming the challenge from modality difference (texts versus graphs) and resolution difference (1-bp versus 100K-bp). We show that our models outperform DeepSEA, DanQ, and a Sei-based model (with local sequence embedding only) in predicting epigenetic profiles of genome sequences on held-out chromosomes. And we analyze the (in)sensitivity of the improvements with regards to the cell line and the resolution associated with the input Hi-C data. We further examine our models’ interpretability in extracting regulatory motifs and found that long-range interactions in chromatin structure data enhanced such interpretability. We last demonstrate utility of our models for predicting noncoding variant effects in gene expression [eQTL (expression quantitative trait locus)] and pathogenicity, using model-predicted epigenetic profile changes as zero-shot or few-shot predictors of variant effects.

## 2 Materials and methods

### 2.1 Data

#### 2.1.1 DNA sequences and epigenetic profiles

We used the same dataset as processed in DeepSEA (Zhou and Troyanskaya 2015). The dataset contains 2.6 million non-overlapping 200-bp bins on either strand (∼17% of the human genome) that is described by a 1K-bp neighborhood encompassing the 200-bp bin at its center and is doubled considering both strands of DNA. Each of the resulting 5.2 million samples is labeled with binary values for 919 epigenetic events associated with 148 cell lines, including binding to 690 TFs from 91 cell lines, 125 DNase I hypersensitive sites from 119 cell lines, and 104 histone marks from eight cell lines. These 919 epigenetic events were curated from ENCODE and Roadmap Epigenomics projects and up to date when DeepSEA was introduced. We also used the same data split as in DeepSEA, that is, chromosomes 8 and 9 (445 thousand samples on both strands, i.e. 227 512 bins on either) for testing, part of chromosome 7 (8000 samples) for validation, and the rest (4.4 million samples) for training. Additional splits completely removing chromosome overlaps were also tested (Supplementary Section S5.4).

We additionally used the updated data from Sei (Chen *et al.* 2022) on our Sei-related models (see Section 2.3). Specifically, we intentionally kept the same training, validation, and testing regions as in DeepSEA, but expanded the description of these 200-bp bins from 1K-bp to 4K-bp neighbors, and expanded the labels from 919 epigenetic events in 148 cell lines to 21 907 in over 1300 cell lines. More details are shown in Supplementary Section S3.3.

#### 2.1.2 Chromatin structures

We additionally used Hi-C data from the ENCODE portal (Luo *et al.* 2020), in the form of interaction frequency matrices, that map genome-wide chromatin interactions. Cell lines GM12878, IMR90, and K562 were related to the 919 epigenetic events. More details are shown in Supplementary Section S1.

### 2.2 Hypothesis tests on the significance of 3D information

For a given cell line’s given replicate, we randomly drew 2% of the samples; partitioned sample pairs with non-zero frequencies into three sets whose sequence similarity percentiles were over, around, or under profile similarity profiles, respectively; and performed one-sided Wilcoxon rank sum test to compare the interaction frequencies of the “under” (or “over”) set and those of the “consistent” set [the null hypothesis being that the former are below (or above) the latter]. The process was repeated three times and the frequencies of *P*-value falling <.05 were collected. More details are shown in Supplementary Section S2.

### 2.3 Machine learning for predicting epigenetic profiles

#### 2.3.1 Architecture

**Figure 1.**
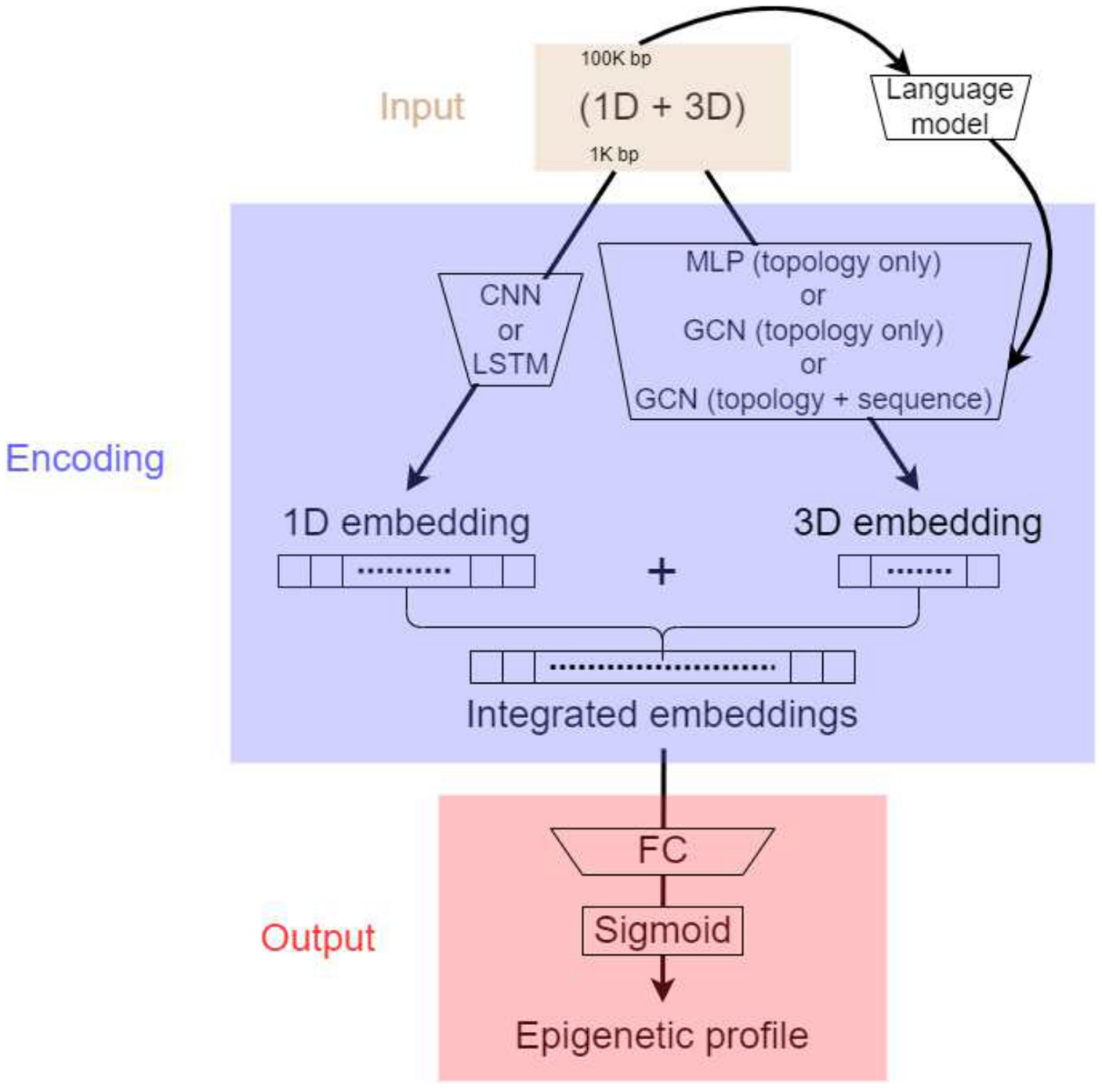
The illustration of our proposed models for epigenetics that can be used for zero-shot or few-shot prediction of noncoding variant effects.

As shown in Fig. 1, our models take two inputs, a 1D local sequence of 1 kb and a 2D global interaction-frequency matrix of the whole genome. The two inputs are separately encoded with different neural networks (to be detailed next) to address a resolution gap—whereas local DNA sequences are available in every base, global chromosome interactions are typically among regions of 100 kb. The resulting embeddings are concatenated and fed to a fully connected layer with 919 neurons and sigmoid activation functions that outputs the probability of each of the 919 epigenetic events. For Sei-related models, the fully connected layer outputs were for 21 907 epigenetic events.

For local sequence embedding (left branch in Fig. 1) of each nucleotide, we used three versions: (i) three layers of 1D CNNs and two layers of max pooling interspersed as in DeepSEA; (ii) one layer of CNN and one layer of max pooling followed by one layer of biLSTM as in DanQ; and (iii) a CNN with both linear and nonlinear paths, followed by residual dilated CNN, spatial basis function transformation and output layers (until the second last layer) as in Sei. Although 1D CNNs are good at extracting and combining local sequence features, biLSTM can capture long-term dependencies. Using a 1D CNN before biLSTM also helps reduce the input size and alleviate the resulting training difficulties for biLSTM. We intentionally kept their architectures the same as DeepSEA, DanQ, and Sei, respectively, to isolate the contribution of chromatin structures. Resulting sequence embeddings were of 925 dimensions for each kilobase (DeepSEA and DanQ) or 15 360 dimensions for each 4Kb (Sei).

For global structure embedding (right branch in Fig. 1) for each chromatin region, we used three versions where the first two consider chromatin topologies only and the last additionally considers DNA sequences. (i) Multi-layer perception (MLP, topology only): depending on the chromatin region (100 kb long for Hi-C of a 100K-bp resolution) that it belonged to, each kb sample was featurized by the corresponding row/column of the chromatin interaction frequency matrix (after normalization) and encoded through a MLP. (ii) GCN (topology only): each kilobase sample was treated as a node in a graph whose node features were simply all ones of 768 dimensions and edge weights were corresponding normalized interaction frequencies. It was encoded through graph convolutional networks or GCN (Kipf and Welling 2016) of three layers. Whereas MLPs can capture global relationship across the chromatin, GNNs including GCNs better capture graph structures and their sparsity. We note that most of the Hi-C data used in the study have <10% nonzero frequencies. (iii) GCN (sequence+topology): we additionally introduced DNA sequences to the GCN (topology only) encoder by replacing the all-one node features with mean-pooled regional sequence embedding from a pretrained DNA language model, DNABERT (Ji *et al.* 2021). More details are provided in Supplementary Section S3.1.

#### 2.3.2 Training

We used binary cross entropy with L1 and L2 regularization as the training loss and optimized hyperparameters including learning rate, the two regularization coefficients, and the choice of replicate per cell line (that provides chromatin structure to encode) over combinations of options. For each hyperparameter combination, part of the models were warm-started with DNA sequence encoders (CNN or CNN/RNN) initialized at the values from pretrained sequence-only models (DeepSEA or DanQ) and trained for up to 40 epochs with early stopping (patience being four epochs). The optimal hyperparameters were chosen based on validation loss, including the optimal Hi-C bioreplicate of each cell line; then each model was trained five times, with the cold-started portion randomly initialized, for performance means and standard deviations. More details are provided in Supplementary Section S3.3.

### 2.4 Interpreting the learned epigenetic predictors

Following DanQ (Quang and Xie 2016), we examined the regulatory significance of the 320 kernels in the first convolutional layer for sequence embedding (kernel size being 8 and 26 bases for CNN and CNN/RNN, respectively). Specifically, for each kernel, the contiguous 8 or 26 bases with the largest activation value were selected for each test sequence and a position-specific frequency matrix (profile) was accordingly built over all test sequences. We queried each of the 320 model-learned profiles against HOCOMOCO (v11 core) (Kulakovskiy *et al.* 2018), a dataset of human transcription-factor binding motifs, using the tool TOMTOM (Bailey *et al.* 2015) with a cutoff E<0.01.

### 2.5 Noncoding variant effect prediction: eQTL

We used the trained epigenetic predictors as featurizers to predict noncoding variant effect on expression levels, i.e. eQTL. We collected cell line specific eQTL data from the Genotype-Tissue Expression project (GTEx Analysis v7): 3845 entries for GM12878 and 11091 for IMR90 using the filter *q*-value no ≤0.05, labeled with whether gene expression levels increase or decrease. These data were relatively label balanced—positive (increase) rates were 49.6% for GM12878 and 49.8% for IMR90. For each entry, pre-trained epigenetic predictors (sequence-based DeepSEA and DanQ as well as our sequence and structure-based models) calculated the differences in predicted probabilities (between the wild type and variants) and the differences in predicted log odds for cell-line epigenetic events (91 for GM12878 and 5 for IMR90 among 919). Using the predicted differences as features, we held out eQTLs from chromosomes 8 and 9 for hyper-parameter tuning when building logistic regression models with L2 regularization (scikit-learn). Through 5-fold cross validation AUROC over the held-out data, we chose the differences in log odds as features and tuned the values for the inverse of regularization strength. After hyper-parameter tuning, we split the rest eQTLs into 5-folds, where each fold contains all eQTLs from three or four chromosomes without chromosome overlaps across folds (sex chromosomes X and Y were kept in the same fold). More details are provided in Supplementary Section S7. As each epigenetic predictor (such as CNN+MLP) was trained five times and used as fixed encoders, the corresponding supervised eQTL classifier was accordingly trained five times to calculate the average AUROC and AUPRC through 5-fold cross validation.

### 2.6 Noncoding variant effect prediction: pathogenicity

We used ncVarDB (Biggs *et al.* 2020), a manually curated noncoding variant dataset, to assess our models’ performance in identifying pathogenic variants. After splicing variants and training variants used by CADD were removed from ncVarDB (see details in Supplementary Section S8.1), 246 pathogenic and 6686 benign variants were retained as the test set (positive rate: 3.55%), 100 variants as the validation set, and 400 variants as the source of the training set that grows from 0 to 400 at the increment of 50, simulating zero-shot (unsupervised) to few-shot (supervised) learning. In the case of zero-shot unsupervised learning where no ncVarDB data are used, we directly used the absolute difference in the predicted epigenetic probabilities between the wild-type (reference) and the alternate (variant) sequences, as a pathogenicity indicator. In the case of few-shot supervised learning, we used the pretrained epigenetic predictors to build a Siamese neural network: two identical genome sequence-structure encoders, one for wild-type and one for variant sequences, whose squared differences of the 919 epigenetic probabilities, magnitude-only and differentiable, are fed to a 919D-to-1D FC layer followed by a sigmoid activation function, to predict whether the given variant is pathogenic or benign. The resulting pathogenicity classifier was trained end to end (unlike the eQTL classifier where a single encoder is used and fixed), using 50–400 training samples, with all parameters warm-started but the last FC layer. The loss function was cross entropy with L2 regularization whose weights were tuned from 1E−12 to 1E−8 log-uniformly and optimized at 1E−11 according to validation loss. As each type of epigenetic predictor (such as CNN+MLP) was trained five times, the corresponding pathogenicity classifier was accordingly initialized and trained five times before an average performance was reported.

## 3 Results

In this section, we first statistically assess the hypothesis that the inconsistency between genome sequence similarity and epigenetic profile similarity can be partly attributed to the chromatin structure. With the hypothesis verified, we adopted it as the rationale of our approach and started with the simplest version of models where chromatin structures are encoded with fully connected layers. We showed that this straightforward way to introduce chromatin structures already outperformed the competing sequence-only models; and confirmed that the model improved the performances by correcting the bias from genome sequence alone using chromatin structure. We also examined the (in)sensitivity of the model to the resolution and the cell line of the input chromatin structure data as well as the splits of the epigenetics-labeled data.

We further evaluate the performances of the more advanced versions where chromatin interactions are modeled as graphs and region sequences as nodes are embedded by DNA language models. These models are more accurate in epigenetic profile prediction than sequence-only models and explainable in extracting regulatory motifs. They are shown to benefit from chromatin interactions as they outperform ablated versions using region orders within chromosomes or chromatin accessibility instead. Importantly, as demonstrated in eQTL and pathogenicity prediction, these models are well-performing unsupervised zero-shot predictors of noncoding variant effects; and they can be flexibly fine-tuned to broadly applicable and accurate few-shot predictors, using few labeled data and no feature engineering or annotation ensembling.

### 3.1 Chromatin structure helps explain a gap between DNA sequence similarity and epigenetic profile similarity

Current machine-learning methods predict epigenetics based on DNA sequences alone, thus leading to similar epigenetics predictions for similar DNA sequences. However, DNA sequence similarity and epigenetic profile similarity are not always consistent and sometimes in disparity. To explain the disparity, we hypothesize that sample pairs with low (high) DNA sequence similarity but high (low) epigenetic profile similarity tend to be closer (farther) in 3D, compared to those with consistent sequence and epigenetic similarities.

**Figure 2.**
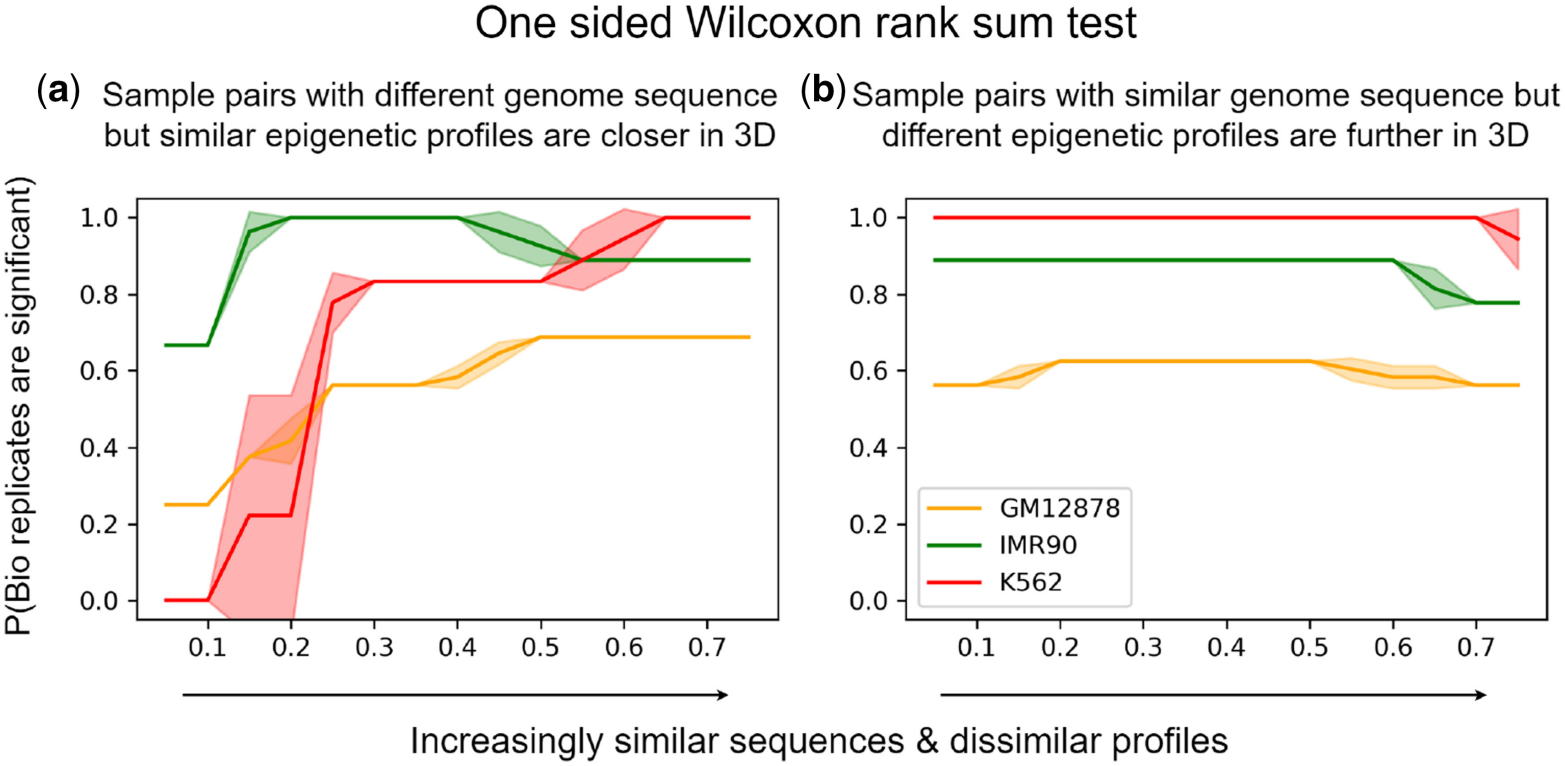
Sequence pairs whose sequence similarity percentiles (a) exceed or (b) lag epigenetic-profile similarity percentiles tend to be closer or further in 3D chromatin structure (across bio-replicates or Hi-C data of chromatin structures for cell lines GM12878, IMR90, and K562), which is frequently found with statistical significance (*P*-value < .05).

One-sided Wilcoxon rank-sum tests validated the hypothesis (Fig. 2) in all but few bio replicates (Hi-C experiment data for chromatin structure). The outliers with no statistical significance included five bio replicates of cell line GM12878 and one bio replicates of cell line IMR90. Most of those bio replicates were also outliers with the most dense interactions (possibly including more spurious weak interactions). More details are provided in Supplementary Section S2.

For sample pairs with low sequence high profile similarity, the trend (as measured by the portions of bio-replicates with statistical significance) was increasing as the sequence–profile disparity (as measured by the cumulative percentage difference or margin between the two similarities) increased. In contrast, sample pairs with high sequence low profile similarity did not see more significance as the disparity increased. The observations agree with biological knowledge: spatial proximity helps explain high profile similarity unexpected from low sequence similarity, as it implies similar chromosome access for epigenetic factors in transcription or modification; but spatial remoteness doesn’t necessarily explain the opposite disparity, as it does not necessarily determine dissimilar chromosome access.

### 3.2 Our models outperform sequence-only epigenetic predictors by filling the gap with chromatin structure

The statistical significance of chromatin structures in addressing the sequence–profile disparity suggests that the additional input of chromatin structure besides DNA sequences would improve epigenetic prediction. We used the chromatin structure data from each of the three cell lines (GM12878, IMR90, and K562) to predict the whole epigenetic profile from 148 cell lines. And we combined three encoders (CNN, CNN/RNN, and residual CNN and B-spline as in Sei) for local DNA sequences and three encoders [MLP, GCN (topology only), and GCN with DNABERT (sequence and topology)] for global chromatin information. Due to label imbalance (positive rate merely 2.06% for the data in DeepSEA/DanQ and 2.32% for the data in Sei) and a focus on top predictions, we report AUPRC (area under the precision–recall curve) in Table 1. Meanwhile, we include AUROC (area under the receiver operating characteristic curve) in Supplementary Table S2 because, unlike AUPRC, AUROC’s baseline is invariant to the portion of positive labels and thus AUROCs can be compared even across different chromatin profiles with different positive portions.

**Table 1.** Epigenetic profile prediction assessed in AUPRC (area under the precision–recall curve) whose base-line value for random classifiers is 0.02.^a^

Method	1D local sequence embedding	3D global structure embedding	AUPRC
DeepSEA	3-layer CNN	N/A	0.342 (Zhou and Troyanskaya 2015)
		MLP (topology only)	0.375 ± 0.004/0.373 ± 0.003/0.370 ± 0.006^b^
Ours	3-layer CNN	GCN (topology only)^c^	0.379 ± 0.002/0.374 ± 0.003/0.377 ± 0.002
		GCN (topology + sequence)^d^	0.380 ± 0.001/0.379 ± 0.002/0.382 ± 0.002
DanQ	CNN+RNN(biLSTM)	N/A	0.371 (Quang and Xie 2016) or 0.358 (reproduced)
		MLP (topology only)	0.379 ± 0.002/0.384 ± 0.003/0.381 ± 0.001
Ours	CNN+RNN(biLSTM)	GCN (topology only)	0.389 ± 0.004/0.389 ± 0.005/0.390 ± 0.002
		GCN (topology + sequence)	0.395 ± 0.003/0.398 ± 0.001/0.393 ± 0.001
Ours (Sei-based)^e^	Residual CNN and B-spline	N/A	0.377 (our reproduced on DeepSEA-selected regions)
		MLP (topology only)	0.410 ± 0.001/0.411 ± 0.000/0.410 ± 0.001
Ours (Sei-based)^e^	Residual CNN and B-spline	GCN (topology)	0.408 ± 0.001/0.409 ± 0.001/0.404 ± 0.001
		GCN (topology + sequence)	0.415 ± 0.000/0.415 ± 0.002/0.418 ± 0.000

a Our models intentionally used the same neural network architectures for DNA local 1D sequence embedding as in DeepSEA, DanQ, or Sei. Thus, their additional introduction of chromatin global 3D structure embedding led to much improved and robust performances.

b Performances using chromatin structure data from the cell line GM12878/IMR90/K562, respectively.

c All-one node features for 100K-bp regions.

d DNABERT-encoded node features for 100K-bp regions.

e Our models with Sei CNN + Spline architecture for local sequence embedding and with/without global structure embedding were on binary classification of 21 907 rather than 919 epigenetic events for local sequence descriptions of 4 kb rather than 1 kb.

All our models incorporating chromatin structures outperformed the baselines DeepSEA, DanQ, and a Sei-based CNN and Spline architecture embedding local sequences alone (Table 1). The most advanced version using GCN with DNABERT to encode chromatin sequences and structures improved AUPRC by over 10% compared to corresponding baselines. We note that the most advanced CNN/RNN+GCN with DNABERT encodes the chromatin’s global sequence and structure together and is complemented by local sequence embedding for desired sensitivity within chromatin regions. The overall improvements were not very sensitive to the input structures’ cell lines especially when GCNs were used. They did slightly decrease (within 0.01) if the resolution of the chromatin structure or the dimension of its embedding decreased (Supplementary Sections S5.1 and S5.2). They were relatively insensitive to parameter initialization and normalization during model training (Supplementary Section S5.3). They were consistent and more pronounced when adopting strictly cross-chromosome data splits (Supplementary Section S5.4). The benefit of node embedding in GCN was consistent whether DNABERT or DeepSEA was used (Supplementary Section S5.6).

**Figure 3.**
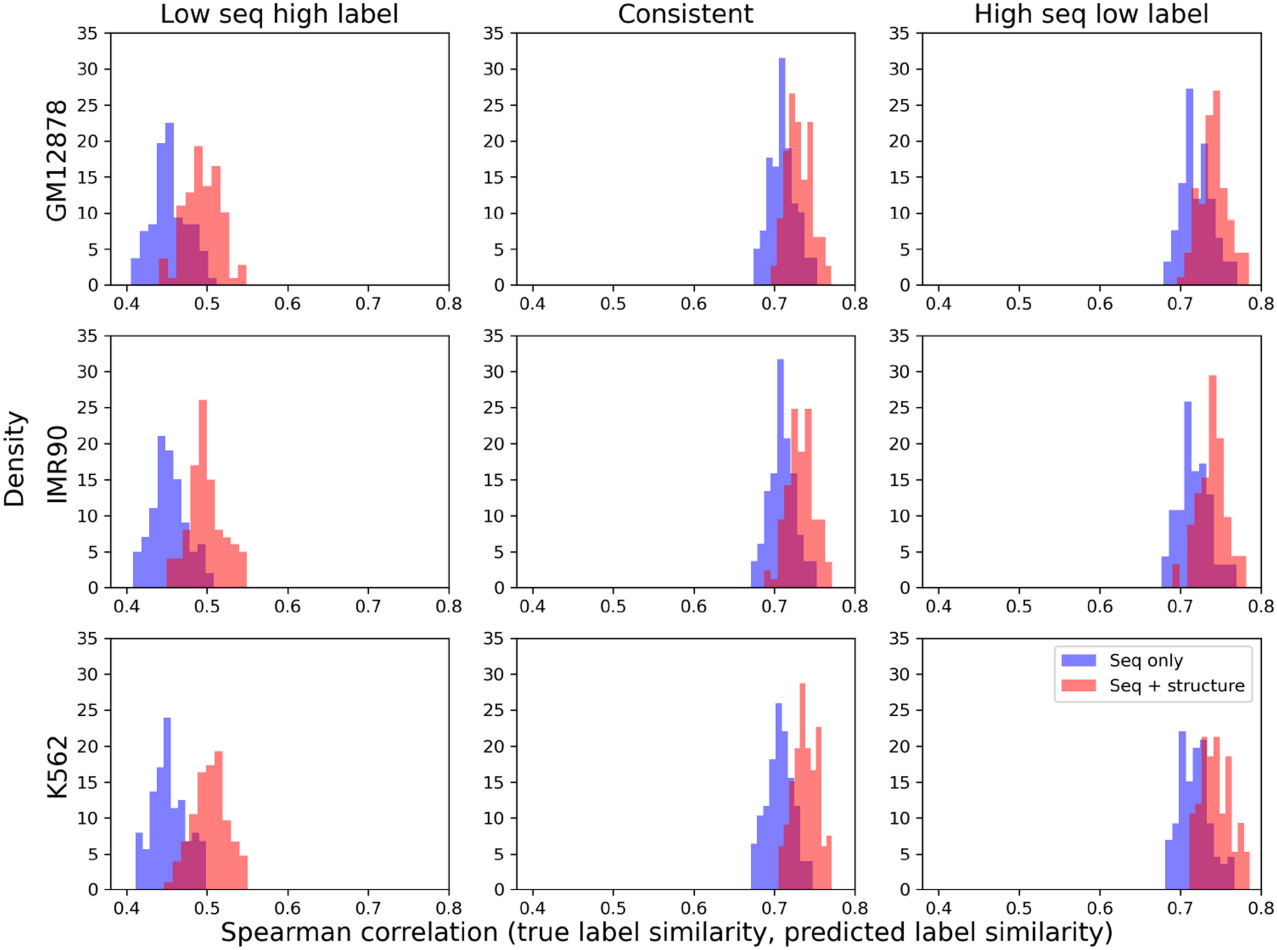
Comparing sequence-only DeepSEA and our sequence+structure basic model, CNN+MLP, in how much they address the disparity between sequence similarity and epigenetic profile similarity.

We next examine the origin of the improvements. To remove possible distraction of model architectures and isolate the contribution of chromatin structures, we compared our basic model, CNN+MLP, to DeepSEA (based on the same CNN for local sequence embedding). We calculated the Spearman correlations between the true and the predicted label similarities for sample pairs randomly picked in the test set (as opposed to random pairs from all the data for hypothesis tests in Section 3.1) and reported the distribution of the Spearman correlations over aforementioned 100 trials in Fig. 3. The chromatin structure information improved the correlation in all three subsets, whether sequence similarity levels were under, consistent with, or over profile similarity levels. And it did the most (+0.05) for DNA pairs whose sequence similarities were significantly under profile similarities, by correcting the sequence-only bias with structure information. Statistical significance of the improvements was supported by one-sided Wilcoxon rank sum tests (Supplementary Section S4.1 including Tables S3–S5).

We further isolate the contribution of 3D chromatin structures by ablation using the input of 1D chromatin regional orders (sequential graphs confined in individual chromosomes) or 1D chromatin accessibility (total read counts or row sums of interaction frequency matrices). As detailed in Supplementary Section 3.2 (methods) and Section S4.3 (results), embedding 3D chromatin structures in CNN+GCN with DNABERT was much better than doing 1D chromatin regional orders in the same model architecture (by 0.02 in AUPRC) and better than doing 1D chromatin accessibilities in MLP (by 0.005–0.010 in AUPRC). Interestingly, embedding 3D chromatin structures in MLP was no better than doing 1D chromatin accessibility in MLP. These results highlight the importance of additionally using and properly embedding 3D chromatin structure data (that contain sparse, long-range interactions). Moreover, additionally including 1D chromatin accessibility embedding could slightly improve AUPRC by around 0.001.

### 3.3 Our models benefit from cell line specificity but tolerate lack thereof

After verifying and tracing the help of chromatin structure (cell line dependent) for epigenetic prediction, we examine whether more help is there for epigenetic events matching the chromatin structure’s cell line or specific types of epigenetic events.

We evaluated AUPRC (AUROC) values for 919 individual epigenetic events’ predictions and reported the percentages of the events where CNN+MLP outperformed DeepSEA (Table 2 and Supplementary Fig. S3). Overall, 89%–94% of all events saw more accurate predictions with the additional input of any chromatin structure (among the three). When the statistics were broken down based on cell line match, matching epigenetic events had ∼6%–9% more frequencies than other events to enjoy such accuracy boost. Therefore, our epigenetic predictors, being cell line specific, benefit from such cell line specificity.

**Table 2.** Percentages of the 919 epigenetic events whose CNN+MLP predictions outperformed DeepSEA (CNN) in AUROC or AUPRC.

	% With better AUROC			% With better AUPRC		
Hi-C cell line	GM12878	IMR90	K562	GM12878	IMR90	K562
Same cell line events	98.02	100	96.79	99.12	96.00	96.05
Other events	92.33	93.84	87.80	92.29	94.15	87.70
All events	92.90	93.88	89.39	92.96	94.16	89.17

**Figure 4.**
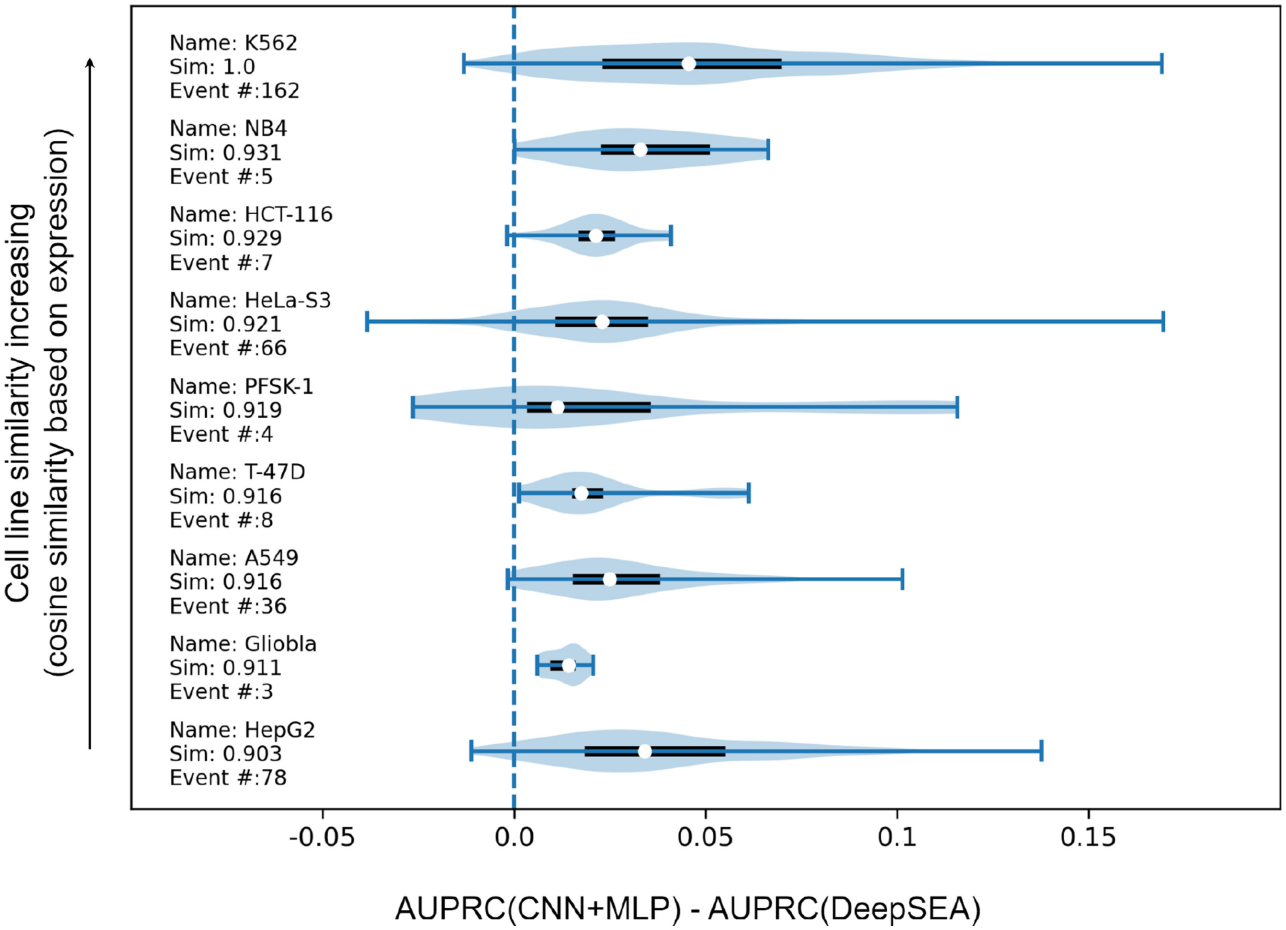
Violin plots of performance boost from DeepSEA to our CNN+MLP (trained with K562 chromatin structure) for epigenetic events of various cell lines. The white dot, the box, and the whiskers indicate the median, the 25%–75% percentile, and the extremes, respectively. The cell lines are arranged in the order of expression similarity to the input K562 cell line, with names, cosine similarities, and numbers of associated epigenetic events given next to corresponding violin plots.

We further examined the AUPRC boost from DeepSEA to our CNN+MLP for the epigenetic events of non-matching cell lines and checked whether the extent of the boost correlated with the degree of cell line similarity. Specifically, we used the CNN+MLP trained with chromatin structure of K562 (ENCFF013TGD) and checked the AUPRC boost for some of the 919 epigenetic events associated with eight more cell lines. Their identities, expression cosine similarity [based on TPM (transcripts per million) values from Dependency Map, https://doi.org/10.6084/m9.figshare.19139906.v1], and the numbers of associated epigenetic events can be found in the texts in Fig. 4, along with violin plots of AUPRC boosts (due to the use of chromatin structure for K562). The violin plots were based on five times the number of associated epigenetic events’ performance differences, because our models were trained in five trials. A weak Spearman correlation of 0.28 was found between median AUPRC boosts and expression similarity to cell line K562, albeit without statistical significance (*P*-value .46).

We also checked the sensitivity of the trained model to the chromatin structure usage. All available chromatin structures, 52 Hi-C experiments from 10 cell lines are collected. We used the pretrained fixed models and made inference on the test set with all available Hi-C. Figure S6 in Supplementary Section S5.5 shows that mismatched cell line’s chromatin structure input didn’t negatively impact the performance for an average epigenetic event, and it did for those events related to the cell line whose chromatin structure was used during training. Nevertheless, either way our basic CNN+MLP performed better than sequence-only DeepSEA when tested with a mismatched cell line’s chromatin structure input.

### 3.4 Our models are interpretable in capturing regulatory motifs

Following DanQ, we analyzed key regions that our models identified over test sequences and compared them to known sequence motifs for TF binding. Of the 320 convolutional kernels of size 26, 164–167 kernels (depending on the cell line of the input chromatin structure) in our basic CNN/RNN+MLP were found to match at least one known sequence motif, which led to 166–168 motifs (see details in Supplementary Section S6.1). Similar numbers were reported in our CNN/RNN+GCN without or with DNABERT. The numbers of matched kernels and identified motifs in our models were similar to those in DanQ (169 matched kernels and 172 motifs).

**Figure 5.**
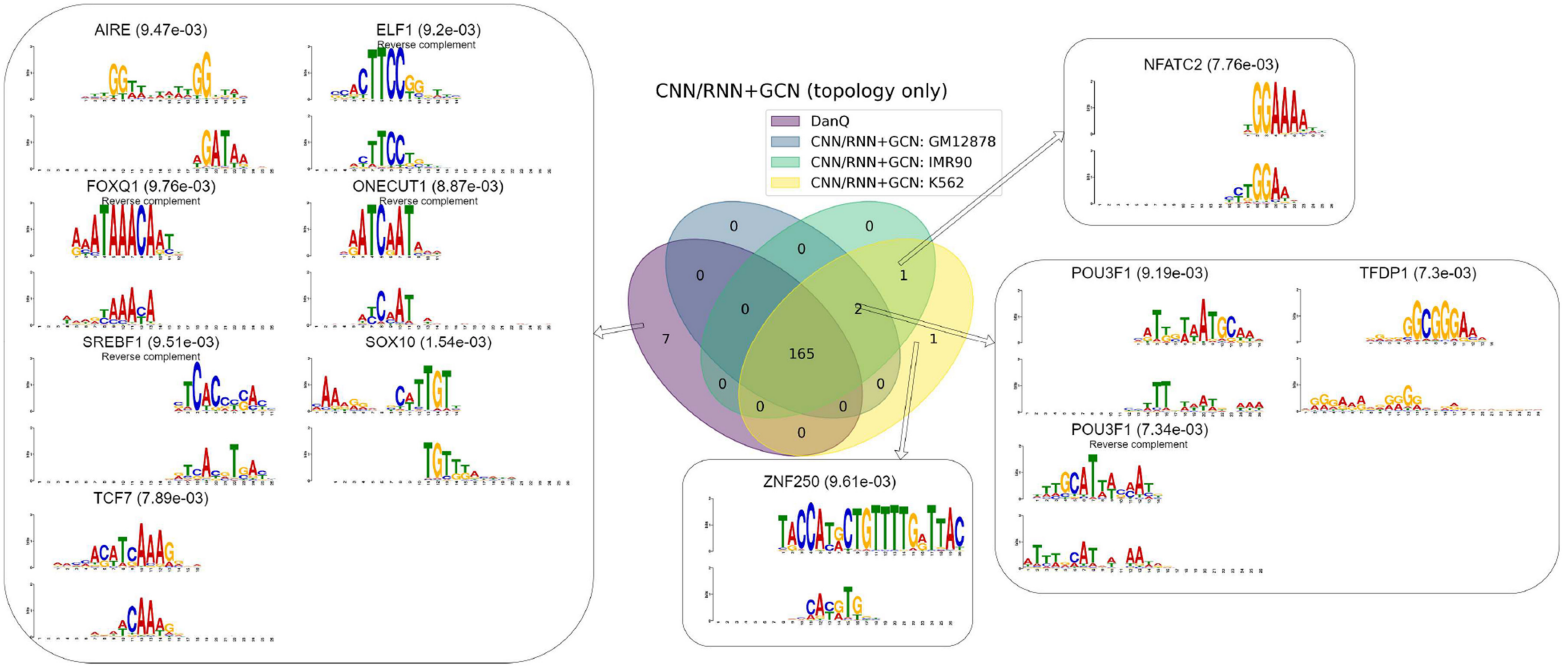
Our CNN/RNN+GCN model identified two motifs (lower right) that were missed by DanQ, regardless of which of the three Hi-C inputs were used. They missed seven motifs (left) that lacked long-range interactions and had low interaction frequencies.

Our chromatin structure-informed models showed complementarity to sequence-only DanQ. When examining motifs identified by CNN/RNN+GCN without DNABERT (thus global chromatin embedding only used topology and not DNA sequences), we found two motifs that were missed by DanQ but consistently identified by our model, regardless of which cell line’s chromatin structure was used. These two motifs are related to genes POU3F1 (both strands) and TFDP1 (Fig. 5). The discovery of these two motifs was attributed to the use of chromatin structure that suggests long-range interactions. Our CNN/RNN+MLP had similar performances. More details are provided in Supplementary Section S6.3.

Meanwhile, seven motifs were found by sequence-based DanQ but missed by all three of our models using CNN/RNN to embed local DNA sequences. Although higher-resolution chromatin structure data such as Micro-C might mitigate the issue, we found that these seven motifs lacked long-range interactions mechanistically and were of lower interaction frequencies compared to other motifs (Supplementary Section S6.4).

### 3.5 Noncoding variant effect prediction: eQTL

We used the aforementioned, pre-trained epigenetic predictors as fixed, feature generators and trained supervised machine learning models to predict noncoding variant effect on gene expression (increase or decrease).

**Figure 6.**
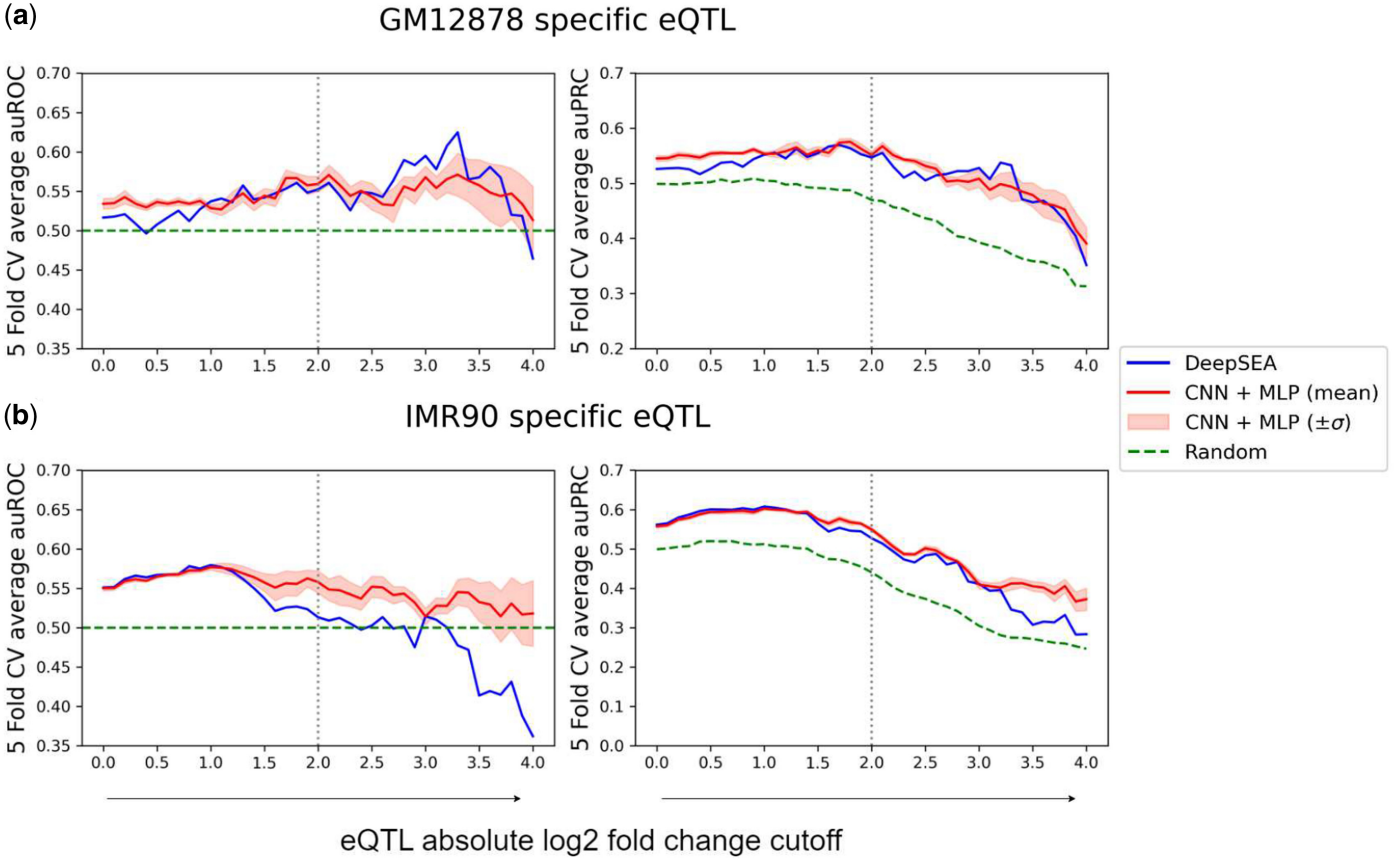
eQTL prediction performances of DNA sequence-only DeepSEA and our chromatin structure-informed CNN+MLP (mean and standard deviation over five trials), at various cutoff of actual expression level fold changes for the test set. Note that as the cutoff of expression change further increases, the number of eQTLs involved further decreases until being too low to support statistical significance. So, our analysis ended at the fold change cutoff of $2^{4}$.

For cell line specific, noncoding eQTL data from GTEx (3845 samples for GM12878 and 11091 for IMR90), cell line specific features (91 for GM12878 and 5 for IMR90) were used to train regularized logistic regression models. Figure 6 shows that, compared to using epigenetic predictions from sequence-only DeepSEA, using those from our basic model to utilize chromatin structure, CNN+MLP, was on average (over five trials) 0.72σ better (in AUROC)/0.63σ better (in AUPRC) for GM12878 and 3.19σ better/4.35σ better for IMR90 (σ is the standard deviation of our model’s performances over five training trials), when focusing on the eQTLs that caused significant expression changes (fold change cutoff at $2^{2}$). We similarly compared epigenetic features predicted by DeepSEA to those by our CNN+GCN with DNABERT and found that similar conclusions held and CNN+GCN with DNABERT (where chromatin structure and sequence were jointly embedded) further improved the margins (Supplementary Fig. S14). We also compared epigenetic predictions by DanQ to those by our CNN/RNN+GCN without or with DNABERT (Supplementary Figs. S15 and S16) and reached similar conclusions. Therefore, the input of chromatin structure improved the prediction of epigenetic events, which further improved eQTL prediction.

### 3.6 Noncoding variant effect prediction: pathogenicity

**Figure 7.**
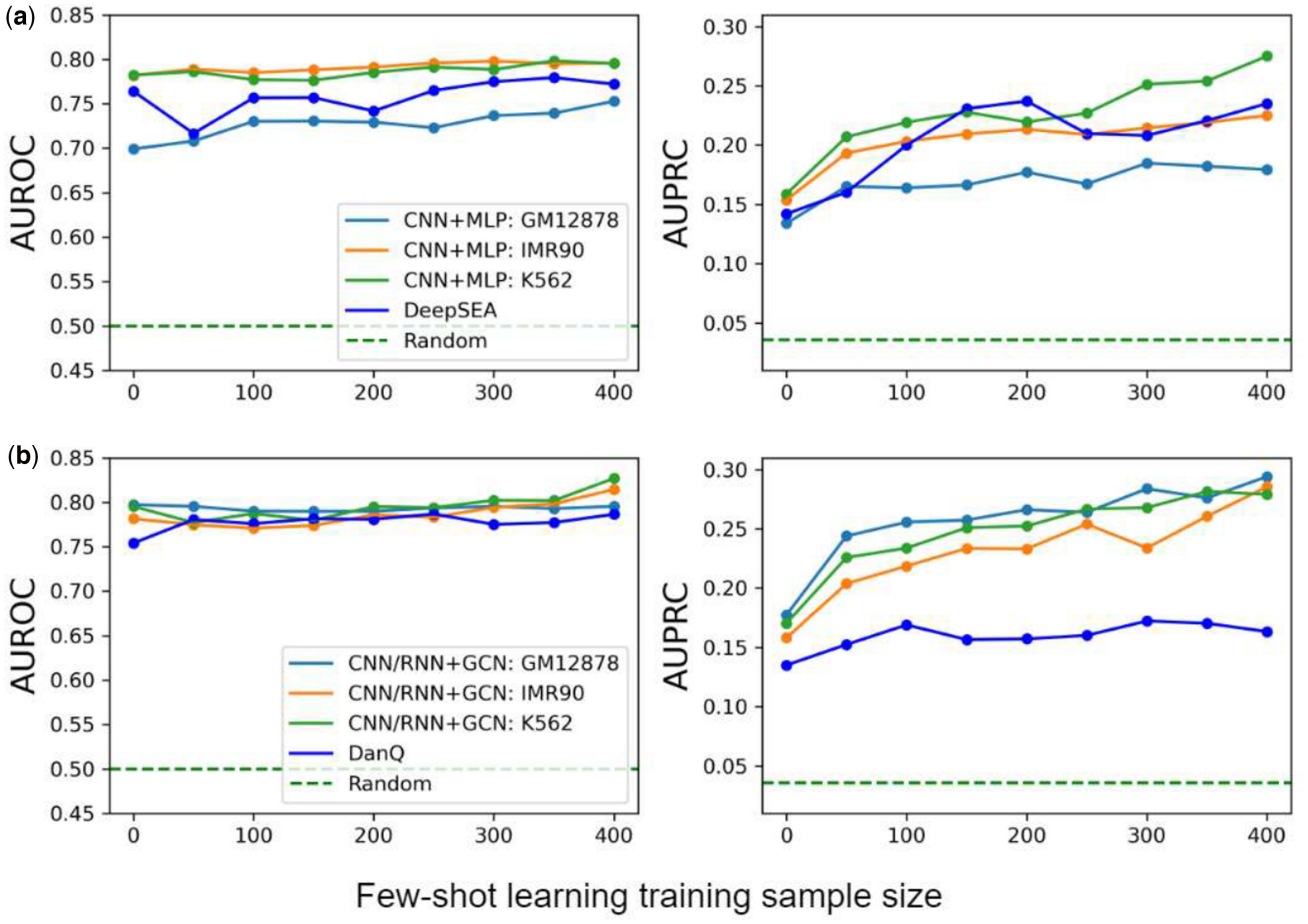
Test performances (AUROC and AUPRC) of zero-shot or few-shot pathogenicity predictors, using (a) CNN+MLP and (b) CNN/RNN+GCN (with DNABERT), improve as the size of the training set increases.

We also tried to assess our models in identifying pathogenic versus benign noncoding variants, whether directly used as an unsupervised “zero-shot” learner or incorporated into a Siamese neural network to be a supervised “few-shot” learner. Figure 7 shows that, even without access to labeled data (variants with known pathogenicity or benign), our zero-shot pathogenicity predictors was of AUROC ∼0.75 and AUPRC ∼0.15. With as few as 100 variants, our few-shot pathogenicity predictors quickly improved the performances as the training data grow and reached AUROC >0.8 and AUPRC >0.25. Such improvements were consistent across model versions or Hi-C data used. The more advanced CNN/RNN+GCN (with DNABERT) showed even more consistency across Hi-C data choices. Numerical values on the unsupervised performances are in Supplementary Section S8.2.

Our noncoding variant effect predictors are broadly applicable to substitutions, insertions, and deletions across various genome positions (except those affecting splicing and resulting in protein sequence variation). In fact, state of the art predictors CADD (Rentzsch *et al.* 2019), DANN (Quang *et al.* 2015), and FATHMM-XF (Rogers *et al.* 2018) did not score for 464 of the 6932 testing variants after splicing variants removed, including those on chromosomes X, Y, and M, all insertion-deletion mutations (indels) as well as some substitutions across various genome positions.

When focusing on the remaining 6468 testing variants, our 400-shot pathogenicity predictors outperformed DANN (in AUROC and AUPRC) and conservation-based PhyloP (Pollard *et al.* 2010) (in AUROC) but were outperformed by CADD and FATHMM-XF that used extensive feature engineering (including other annotations) and much more training data (up to tens of millions) (Supplementary Tables S24 and S25). Meanwhile, our predictors, free of feature engineering and annotation ensembling and trained on few labeled variants, complement those ensemble methods and can improve the latter even using simple average predictions (Supplementary Table S26). More details are provided in Supplementary Section S8.3.

## 4 Conclusion

Toward noncoding variant effect prediction, we have built multi-modal machine learning models that use both genome sequences and chromatin structures to predict epigenetic profiles, whereas previous methods only used genome sequences. To rationalize the approach, we have verified that there is often a disparity between sequence similarity and epigenetic-profile similarity and such disparity can be explained by, thus mitigated by, spatial proximity. Accordingly, we have used the combination of various neural networks to embed local 1D kilobase sequences (CNN and CNN/RNN) and global 3D chromosome structures represented in 2D graphs (MLP and GCN), that are available in different resolutions, and concatenated the embeddings to feed epigenetic prediction layers. We have additionally introduced a version where the two embedding processes are coupled, by using a pretrained DNA language model to embed 100K-long bases as node features before embedding the 2D graphs of chromatin structures. Compared to sequence-only methods, our models robustly improve the accuracy of epigenetic prediction and complement the explainability in extracting regulatory motifs.

For noncoding variant effect prediction, these pretrained models are used directly as unsupervised zero-shot predictors or incorporated into a Siamese neural network as supervised few-shot predictors, as demonstrated in eQTL and pathogenicity prediction. Without manual feature engineering or annotation ensembling, our models are shown to be fine-tunable for various effects and broadly applicable to variants of various types at various genome positions. They are also shown to complement and boost annotation-based pathogenicity predictors.

## Supplementary Material

btad541_Supplementary_Data
