# Supplementary material for: Multimodal learning of noncoding variant effects using genome sequence and chromatin structure
Source: Bioinformatics. 2023 Sep 5;39(9):btad541. doi: 10.1093/bioinformatics/btad541 (PMC10502240; doi:10.1093/bioinformatics/btad541)
Supplement: btad541_Supplementary_Data [file btad541_supplementary_data.pdf]

Multimodal learning of noncoding variant effects using genome sequence and chromatin structure
(Supplementary Information)

Wuwei Tan and Yang Shen
Texas A&M University

August 2023

1 Hi-C Experiment Data Summary

We downloaded Hi-C data in August 2022 from the ENCODE portal [1] using the following filters of experiment search — organism: 'Homo sapiens', perturbation: 'not perturbed', genome assembly: 'hg19' (as in DeepSEA's data), and available file types: 'hic'. Out of ten resulting cell lines, three were related to the 919 epigenetic events, namely GM12878, IMR90 and K562. With these cell lines chosen for the biosample filter, we downloaded corresponding Hi-C data with quality status as 'released' and output type as 'mapping quality threshold chromatin interactions', including 16, 9, and 6 bio replicates (experiments) for GM12878, IMR90 and K562, respectively. We used the 100K-bp resolution by default and also obtained that of 500K and 1M bps for analyses.

There were 52 Hi-C experiments from 10 cell lines as of the end of August 2022 as follows:

- Endothelial cell of umbilical vein: ENCFF606XNW
- **GM12878**: ENCFF014VMM, ENCFF053BXY, ENCFF065LSP, ENCFF223UBX, ENCFF227XJZ, ENCFF355OWW, ENCFF473CAA, ENCFF482LGO, ENCFF514XWQ, ENCFF563XES, ENCFF632MFV, ENCFF688KOY, ENCFF718AWL, ENCFF777KBU, ENCFF799QGA, ENCFF812THZ
- GM23248: ENCFF768UBD
- HAP1: ENCFF230HVV
- hTERT RPE-1: ENCFF922ERE
- **IMR90**: ENCFF029MPB, ENCFF043EEE, ENCFF303PCK, ENCFF366ERB, ENCFF894GLR, ENCFF920CJR, ENCFF928NJV, ENCFF997RGL, ENCFF999YXX
- KBM-7: ENCFF239BHZ, ENCFF277LAN, ENCFF397CMD, ENCFF698KFV, ENCFF945TUH
- Keratinocyte: ENCFF349RZY, ENCFF406KJN, ENCFF569RJM, ENCFF738YON
- **K562**: ENCFF013TGD, ENCFF097SKJ, ENCFF406HHC, ENCFF464KRA, ENCFF929RPW, ENCFF996XEO

- Mammary epithelial cell: ENCFF198SSL, ENCFF251UEF, ENCFF307PDL, ENCFF491AOR, ENCFF543USQ, ENCFF706SFK, ENCFF773ITV, ENCFF942LTN

Bold-faced are three cell lines associated with some of the 919 epigenetic events to predict.

The Hi-C data used are in the form of interaction frequency matrices. Higher interaction frequencies in the 2D data indicate closer proximity in 3D. The raw interaction frequency matrices were normalized by adding one's to its diagonal elements and then dividing each element by the square root of the product of the row sum and the column sum [2].

2 Hypothesis Tests on the Significance of 3D Information

2.1 Subsets of sequence pairs defined by sequence–profile disparity

For a given pair of genome sequences $\mathbf{x}_i$ and $\mathbf{x}_j$ (of lengths $L = 1000$ in our study), the sequence similarity is defined as $\mathrm{SIM}_{\mathrm{seq}}(\mathbf{x}_i, \mathbf{x}_j) = \frac{1}{L}\sum_{l=1}^{L} \mathbb{1}\{x_i^l = x_j^l\}$ where x_i^l (x_j^l) is the categorical type of the l-th nucleic acid in the sequence $\mathbf{x}_i$ ($\mathbf{x}_j$). For a given pair of epigenetic profiles (labels) $\mathbf{y}_i = \mathbf{y}(\mathbf{x}_i)$ and $\mathbf{y}_j = \mathbf{y}(\mathbf{x}_j)$ (of 919 dimensions in our study) for sequences $\mathbf{x}_i$ and $\mathbf{x}_j$, the epigenetic profile similarity is defined as $\mathrm{SIM}_{\mathrm{epigen}} = \frac{1}{919}\sum_{k=1}^{919} \mathbb{1}\{y_i^k = y_j^k\}$ where y_i^k (y_j^k) is the binary value for the k-th epigenetic event in the profile $\mathbf{y}_i$ ($\mathbf{y}_j$).

Within a selected set of sequence pairs (among 2% random samples of sequences we chose all pairs with non-zero normalized interaction frequencies), we calculated the cumulative percentage (percentile rank) for the sequence similarity and that for the profile similarity, for each pair $\mathbf{x}_i$ and $\mathbf{x}_j$: $\mathrm{CumuPct}(\mathrm{SIM}_{\mathrm{seq}}(\mathbf{x}_i, \mathbf{x}_j))$ and $\mathrm{CumuPct}(\mathrm{SIM}_{\mathrm{epigen}}(\mathbf{x}_i, \mathbf{x}_j))$. For instance, if a given pair has similarity above 30% of all pairs considered, then its cumulative percentage is 30. Accordingly, among all pairs considered, the subsets where sequence similarity is under, around, and over profile similarity are defined as:
$\{(\mathbf{x}_i, \mathbf{x}_j)|\mathrm{CumuPct}(\mathrm{SIM}_{\mathrm{seq}}(\mathbf{x}_i, \mathbf{x}_j)) \leqslant \mathrm{CumuPct}(\mathrm{SIM}_{\mathrm{epigen}}(\mathbf{x}_i, \mathbf{x}_j)) - \delta\}$,
$\{(\mathbf{x}_i, \mathbf{x}_j)|\mathrm{CumuPct}(\mathrm{SIM}_{\mathrm{epigen}}(\mathbf{x}_i, \mathbf{x}_j)) - \delta \leqslant \mathrm{CumuPct}(\mathrm{SIM}_{\mathrm{seq}}(\mathbf{x}_i, \mathbf{x}_j)) \leqslant \mathrm{CumuPct}(\mathrm{SIM}_{\mathrm{epigen}}(\mathbf{x}_i, \mathbf{x}_j)) + \delta\}$, and
$\{(\mathbf{x}_i, \mathbf{x}_j)|\mathrm{CumuPct}(\mathrm{SIM}_{\mathrm{seq}}(\mathbf{x}_i, \mathbf{x}_j)) \geqslant \mathrm{CumuPct}(\mathrm{SIM}_{\mathrm{epigen}}(\mathbf{x}_i, \mathbf{x}_j)) + \delta\}$, respectively. δ is a margin parameter. The "under" and "over" subsets were also referred to as low-sequence high-profile similarity and high-sequence low-profile similarity, respectively.

2.2 Data visualization

We illustrate the three subsets for the interaction frequency data, using the example of Hi-C experiment ENCFF014VMM for cell line GM12878, the same replicate chosen my our machine learning models for the cell line (see details in the next section). In Figure S1 each pair is shown as a point in the 2D space of cumulative percentages in sequence and profile similarities, and colored with the cumulative percentages in normalized interaction frequencies. Visual inspection indicated that the "low-sequence high-profile similarity" ("under") region was enriched with higher interaction frequencies (closer proximity).

2.3 Wilcoxon rank-sum tests

To statistically compare those pairs with low-sequence high-profile similarity (or high-sequence low-profile similarity) and those pairs with sequence–profile consistent similarities, one-sided Wilcoxon

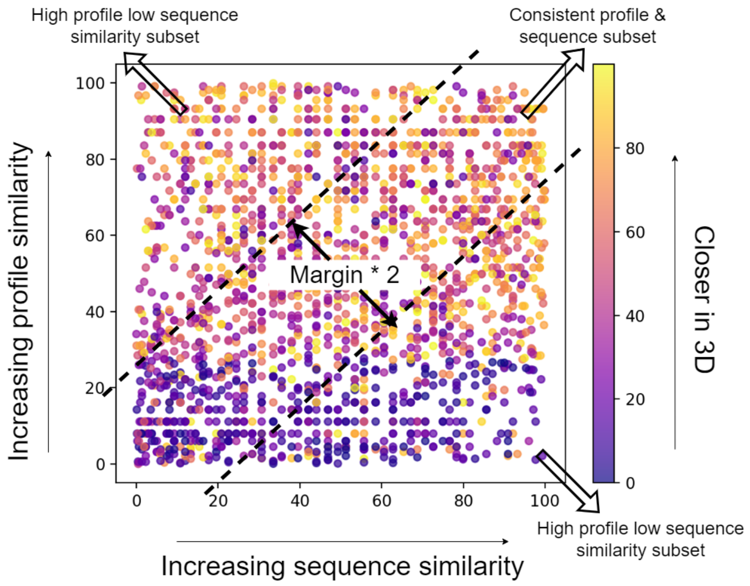

Figure S1: Three subsets of sequence pairs, defined by the disparity between their percentiles in sequence similarity (x-axis) and percentiles in epigenetic profile similarity (y-axis), show distinct patterns in their 3D proximity / normalized interaction frequencies (colored in cumulative percentage). Sequence pairs whose percentiles in epigenetic profile similarity significantly surpass their percentiles in sequence similarity (upper left corner) tend to be enriched with closer proximity (brighter color).

rank-sum tests were employed, following 3 trials of initial 2% samples, for each Hi-C data corresponding to a replicate (experiment) of a given cell line. The null hypotheses were that the normalized interaction frequencies of the low-sequence high-profile similarity (high-sequence low-profile similarity) subset were no higher (lower) than those of the consistent sequence-profile similarity subset. We evaluated how often p-values were below 0.05 among the Hi-C replicates, then calculated the mean and the standard deviation across 3 trials. Because of the daunting number of pairs (over 3 trillion) among 2.6 million original samples, the sampling rate 2% was adopted to reach a billion pairs, which cost 6 hours of pairwise similarity calculation and Wilcoxon rank-sum test and 8GB of NumPy array per trial.

From Figure 2 of the main text we found that all but few replicates showed statistical significance of dominant frequencies especially when the separating margins increased, supporting that those pairs with exceptionally low (high) epigenetic profile similarities compared to their sequence similarities tended to be further (closer) in 3D.

2.4 Outliers

We traced some origins of the few outliers in the Wilcoxon rank sum tests above. First, we examined the sparsity of the normalized interaction frequencies for all involved Hi-C replicates by defining sparsity as the portion of the zero elements in a normalized interaction frequency matrix. We found that the five Hi-C replicate outliers for cell line GM12878 were of lower sparsity compared to most other replicates for the same cell line (Figure S2 left). However this was not the case for cell line IMR90. For sequence pairs sampled in all GM12878 replicates we further calculated the Spearman correlation between the sparsity and the Wilcoxon rank sum test statistic (the distance

between empirical distribution functions), as a function of sequence–profile disparity margin δ. We found that the correlations were positive and high for both "under" and "over" subsets (Figure S2 right), supporting that low sparsity for this cell line contributes to low statistical significance. The "over" subset (high sequence low profile/label similarity) seemed to be more sensitive to sparsity, judging by its higher Spearman correlations. Meanwhile, the Spearman correlation for the "under" subset increased as δ increased, showing that the standing out interactions dominated the sparsity–disparity relationship for these sequences. So if the low sparsity was partially due to spurious weak interactions misidentified in experiments, increasing margin tended to spot the standing-out, strong interactions more. In contrast, the Spearman correlation for the "over" subset was relatively stable as δ changed.

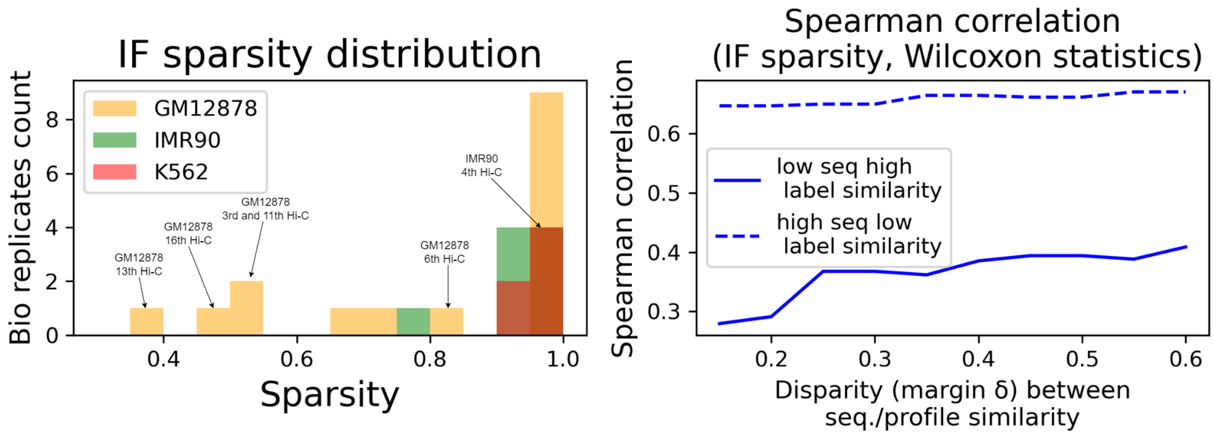

Figure S2: (Left) The histograms of Hi-C experiment data's 3D interaction frequency sparsity levels. Arrow pointed are few outliers found in Wilcoxon rank sum tests and they tend to be of low sparsity for cell line GM12878. (Right) For sequence pairs sampled for cell line GM12878, the Spearman correlation between 3D interaction sparsity and Wilcoxon rank sumt test statistic was positive and high for both "under" (low sequence high label similarity) and "over" (high sequence low label similarity) and it increased for the "under" subset as the sequence–profile disparity margin δ increased. The results suggest that, as epigenetic profile similarity exceedingly surpasses sequence similarity, Hi-C data of less non-zero interaction frequencies possibly due to reduced spurious weak interactions would better reveal such disparity.

3 Machine Learning for Epigenetic Profile Prediction

3.1 Model architectures

Local DNA sequence embedding. To encode local DNA sequences (kilobases) we intentionally used the model architectures from state-of-the-art sequence-only epigenetic predictors as follows.

CNN as in DeepSEA [3]:

1. Convolution layer (320 kernels, kernel size: 8, step size 1) with ReLU
2. Max pooling layer (kernel size 4, step size 4)
3. Dropout (20 percent)

4. Convolution layer (480 kernels, kernel size 8, step size 1) with ReLU
5. Max pooling layer (kernel size 4, step size 4)
6. Dropout (20 percent)
7. Convolution layer (960 kernels, kernel size 8, step size 1) with ReLU
8. Dropout (50 percent)
9. Fully connected layer (925 neurons)

CNN/RNN as in DanQ [4]:

1. Convolutional layer (320 kernels, kernel size 26, step size 1) with ReLU
2. Pooling layer (Window size 13, step size 13)
3. Dropout (20 percent)
4. BiLSTM layer (containing 320 hidden features)
5. Dropout (50 percent)
6. Fully connected layer (925 neurons)

Global chromatin structure embedding. We additionally used the following three architectures to embed chromatin regions (100 kilobases) where each kilobase belongs.

MLP (topology only):

1. Fully connected layer (1000 neurons) with ReLU. The input dimension is varies with the chromatin structure resolution – 30971 for 100K bp, 6207 for 500K bp and 3114 for 1M bp.
2. Fully connected layer (400 neurons) with ReLU
3. Fully connected layer (128 neurons) with ReLU

GCN (topology only):

1. GCN layer (input node feature [all-ones] dimension 768, output node feature dimension 1000) with ReLU.
2. Dropout (20 percent)
3. GCN layer (output node feature dimension 400)
4. Dropout (20 percent)
5. GCN layer (output node feature dimension 128)

GCN (sequence + topology):

1. GCN layer (input node feature [mean-pooled DNABERT over 497 tokens first and then over all 500-bp windows] dimension 768, output node feature dimension 1000) with ReLU.
2. Dropout (20 percent)
3. GCN layer (output node feature dimension 400)
4. Dropout (20 percent)
5. GCN layer (output node feature dimension 128)

We used DNABERT to embed each 100K-bp with a 500-bp sliding window and a 250-bp stride by averaging the 768-dimensional embeddings of all windows. For each window, we used 497 tokens (495 6-mers plus special tokens CLS for sentence class and SEP as sentence separator), obtained a 768 by 497 dimensional embedding, and used the row average to mitigate the impact of the starting position.

Epigenetic profile prediction (Output). Based on aforementioned local sequence and global structure embedding, we predict the probability of each event in the epigenetic profile, using the following architecture.

1. Concatenation layer to combine the local sequence embedding of a kilobase (925 dimensions) and the global chromatin structure embedding of the 100 kilobases that it belong to (128 dimensions) followed by ReLU
2. Fully connected layer (919 neurons) with sigmoid

3.2 Ablated models' architectures: replacing or appending chromatin structure with chromatin accessibility

To test the usefulness of the chromatin structure information as an input, we also tested an input of chromatin accessibility. We used the total number of reads per genomic location from the Hi-C data, in other words, row sum of the un-normalized interaction frequency matrices, to indicate chromatin accessibility. We then embedded chromatin accessibility as follows.

MLP (chromatin accessibility only)

1. Fully connected layer (16 neurons) with ReLU. The input dimension is 1 (aforementioned total reads / row sums after being divided by the average total read / row sum)
2. Fully connected layer (64 neurons) with ReLU
3. Fully connected layer (128 neurons in v1 and 64 neurons in v2) with ReLU

Besides replacing chromatin structure information to showcase the latter's usefulness, chromatin accessibility can also append chromatin structure information to complement the latter.

Global chromatin structure + chromatin accessibility embedding. The output of this embedding module would be concatenated with the output of the local sequence embedding module (925 dimensional) and then fed to the output module (see Sec. 3.1).

MLP (normalized IF and chromatin accessibility): Concatenate the input from the normalized interaction frequency (IF) matrix (30,971-dimensional) and that from the normalized row sum, then feed the concatenated input to 3-layer MLPs of 1000, 400, and 128 neurons, respectively, with ReLU (the same as MLP (topology only).

MLP (normalized IF embedding and chromatin accessibility embedding v1/v2): Concatenate the embedding output of MLP (topology only) and that of MLP (chromatin accessibility v1/v2).

GCN (topology + sequence) + MLP (chromatin accessibility): Concatenate the embedding output of GCN(topology+sequence) and MLP (chromatin accessibility) (v1).

3.3 Sei-based models and data

Besides DeepSEA-based architecture to embed local DNA sequence and data for epigenetic prediction, we also used Sei-based architecture and data for a series of Sei-based models.

Local DNA sequence embedding. Compared to DeepSEA, Sei's model architecture consists of three sections: (1) a CNN with both linear and nonlinear paths; (2) residual dilated CNN; and (3) spatial basis function transformation and output layers. More details can be found in [5]. We used the output of Sei's second last layer (15360-dimensional) as its local DNA sequence embedding that would be concatenated with the output of global chromatin structure embedding modules (128-dimensional) and fed to an output module (see Sec. 3.1). The output dimension was 21,907 TF binding, histone marks and DNA accessibility events rather than 919.

Data. So that we can compare Sei-based models to our other models, we processed/converted Sei's data as follows. Except for the labels expanded from 919 to 21,907 epigenetic events and the input / 200-bp region description expanded from 1K to 4K-bp neighbors, we tried to keep the rest of the data the same as DeepSEA. Therefore, we converted Sei proposed-epigenetic profile, from genome assembly GRCh38/hg38 to GRCh37/hg19. Then, for each DeepSEA selected 200-bp region (now described by surrounding 4 kilobases), as Sei had suggested), we assigned it with a positive label for a specific epigenetic event if the center bp overlaps with a peak of the event.

3.4 Model training

The loss function includes binary cross entropy as well as L1 (LASSO) and L2 (Ridge) regularization. Binary cross entropy was averaged over all training samples in a given batch, positive (sense) or negative (antisense) strand, that were essentially treated independent. L1 regularization was on parameters of the chromatin structure encoder (right branch of Figure 1 in the main text) and those of the output fully connected layer; and L2 regularization was on all parameters of each model.

We used the Adam optimizer with default weight decay schedule to train our models, using data in batches of size 512, for up to 40 epochs unless early stopping criteria is met (see below). We initialized the DNA-sequence encoders not randomly but from pre-trained DeepSEA and DanQ for CNN and CNN/RNN respectively. The pre-trained DeepSEA was directly downloaded from the original publication's shared data; and the pre-trained DanQ was reproduced in PyTorch by ourselves (the original release was in Keras). The other parameters were initialized randomly and the impact of these parameters' initialization was studied in Sec. S5.3. Early stopping criteria is a patience of 4 epochs (40 validations, see below) based on the validation loss. In other words, before reaching the maximum epochs, training would stop if no decrease is observed for validation loss in four consecutive epochs. The checkpoint with the lowest validation loss was chosen to define optimal parameters of our models.

We trained our models on single A100 GPUs. Training per epoch took around 20 minutes, 20–25 minutes, and 4 hours for CNN+MLP, CNN/RNN+MLP, and CNN+GCN (or CNN/RNN+GCN) respectively. GCN-based chromatin embedding took much longer time due to the fact that message passing over the entire chromatin graph was repeated for each batch, which can be sped up. For Sei-based models, it took 4 hours and 6 hours for Sei+MLP and Sei+GCN, respectively, due to the 4-times longer local sequences and over 20-times more binary classification tasks.

Our hyperparameters include the initial learning rate for the optimizer Adam, the coefficients of L1 and L2 regularizations (only L2 was used for Sei-based models to save training time), and the bio replicate (defining which input to feed the chromatin structure encoder) for a given cell

line. For each input Hi-C replicate option for the three cell lines described in Sec. S1, we performed grid search over these hyperparameters ($lr \in$ {1E-2, 5E-3, 1E-3, 5E-4, 1E-4, 5E-5, 1E-5}, L1 coefficient $\in$ {1E-6, 1E-7, ..., 1E-12}, L2 coefficient $\in$ {1E-6, 1E-7, ..., 1E-12}) and trained model parameters for each hyperparameter combination. We chose the optimal hyperparameters based on the validation loss averaged over all input Hi-C replicates. We calculated validation loss 10 times equi-spaced during each epoch, each time using the incremental 10% training data to train and all validation data to validate. The tuned hyperparameters can be found in Table S1. With the tuned hyperparameters, we chose the optimal replicates for each cell line: in the CNN+MLP model they were ENCFF014VMM for cell line GM12878, ENCFF928NJV for cell line IMR90, and ENCFF013TGD for cell line K562; and the same replicates were used for our other models.

Table S1: Tuned hyperparameters including learning rate and regularization weights.

	Learning rate	L1 weight	L2 weight
CNN+MLP	5E-5	1E-11	1E-8
CNN+GCN w/ all-ones	5E-5	1E-12	1E-8
CNN+GCN w/ DNABERT	5E-5	1E-12	1E-8
CNN+GCN w/ DNABERT (binary graph)	1E-4	1E-11	1E-9
CNN/RNN+MLP	5E-5	1E-11	1E-9
CNN/RNN+GCN (topology only)	5E-5	1E-12	1E-8
CNN/RNN+GCN w/ DNABERT	1E-4	1E-12	1E-8
Ours (with Sei CNN + Spline architecture)	1E-5	N/A	1E-11
Our Sei+MLP	1E-5	N/A	1E-7
Our Sei+GCN w/ DNABERT	1E-5	N/A	1E-10
CNN+MLP (resolution 500K bp)	5E-5	1E-11	1E-10
CNN+MLP (resolution 1M bp)	5E-5	1E-12	1E-10
CNN+MLP (embedding 64)	5E-5	1E-12	1E-10
CNN+MLP (embedding 256)	5E-5	1E-11	1E-8
CNN+MLP (embedding 512)	5E-5	1E-11	1E-10
CNN+MLP (unnormalized IF only)	1E-4	1E-11	1E-10
CNN+MLP (chromatin accessibility only)	5E-5	1E-11	1E-9
CNN+MLP (normalized IF and chromatin accessibility)	5E-5	1E-11	1E-9
CNN+MLP (normalized IF)+MLP (chromatin accessibility)(default: 128-d)	1E-5	1E-11	1E-9
CNN+MLP (normalized IF)+MLP (chromatin accessibility) (64-d)	5E-5	1E-12	1E-10
CNN+GCN (topology + sequence)+MLP (chromatin accessibility)	5E-5	1E-12	1E-11
CNN+GCN (sequential topology + sequence)	5E-5	1E-12	1E-9

With hyperameters tuned, each of our models was trained five times with random initialization (see more details below) and used to make inference on the test set. Following DeepSEA, our trained models predicted probabilities of epigenetic events for each pair of samples corresponding to the same 1K-bp on two strands and took the average as the final prediction for the 1K-bp. AUPRC and AUROC were calculated over all testing 1K-bp's and their mean and standard deviation over 5 training repeats were reported for each model.

3.5 Performance comparison

Besides using the major assessment metric of AUPRC (Area Under the Precision-Recall Curve) in Table 1 of the main text, we also used another metric of AUROC (Area Under the Receiver Operating Characteristic curve) to compare our models' performances in epigenetic profile prediction with the state of the art (DeepSEA and DanQ). Unlike AUPRC, AUROC's baseline is invariant to the portion of positive labels and thus AUROCs can be compared even across different chromatin

profiles with different positive portions. We intentionally used the same neural network architectures for DNA local 1D sequence embedding as those in DeepSEA and DanQ, so that performance margins can be attributed surely to the newly added chromatin global 3D structure embedding. The same conclusion can be made when comparing our Sei-based models without and with global structure embedding.

Table S2: Epigenetic profile prediction assessed in AUROC (Area Under the Receiver Operating Characteristic curve) whose base-line value for random classifiers is 0.50. Our models intentionally used the same neural network architectures for DNA local 1D sequence embedding as in DeepSEA, DanQ, or Sei, and their additional introduction of chromatin global 3D structure embedding led to improved and robust performances. [1]Performances using chromatin structure data from the cell line GM12878 / IMR90 / K562, respectively. [2]All-one node features for 100K-bp regions. [3]DNABERT-encoded node features for 100K-bp regions. [4]Our models with Sei CNN + Spline architecture for local sequence embedding and without/with global structure embedding were on binary classification of 21,907 rather than 919 epigenetic events for local sequence descriptions of 4 kilobases rather than 1 kilobase.

Method	1D Local Sequence Embedding	3D Global Structure Embedding	AUROC
DeepSEA	3-layer CNN	N/A	0.933 [3]
		MLP (topology only)	0.941 ± 0.001 / 0.940 ± 0.000 / 0.939 ± 0.002
Ours	3-layer CNN	GCN (topology only)[2]	0.942 ± 0.001 / 0.940 ± 0.001 / 0.942 ± 0.001
		GCN (topology + sequence)[3]	0.942 ± 0.000 / 0.942 ± 0.000 / 0.943 ± 0.001
DanQ	CNN+RNN(biLSTM)	N/A	0.938 [4] / 0.930 (reproduced)
		MLP (topology only)	0.938 ± 0.000 / 0.939 ± 0.001 / 0.939 ± 0.001
Ours	CNN+RNN(biLSTM)	GCN (topology only)	0.940 ± 0.001 / 0.940 ± 0.001 / 0.941 ± 0.000
		GCN (topology + sequence)	0.941 ± 0.001 / 0.942 ± 0.000 / 0.941 ± 0.000
Ours (Sei-based)[4]	Residual CNN and B-spline	N/A	0.925 (our reproduced on DeepSEA selected regions)
		MLP (topology only)	0.941 ± 0.000 / 0.941 ± 0.000 / 0.941 ± 0.000
Ours (Sei-based) [4]	Residual CNN and B-spline	GCN (topology only)	0.938 ± 0.000 / 0.939 ± 0.000 / 0.936 ± 0.000
		GCN (topology + sequence)	0.937 ± 0.000 / 0.937 ± 0.001 / 0.939 ± 0.000

4 Tracing the origin of improvements by comparing DeepSEA (sequence-only) and our CNN+MLP (sequence + structure)

In an effort to trace the origin of our models' improvements and verify the contribution of our models' rationale (tested in Section S2), we examined which subset of test samples or sample pairs benefited more compared to others. We compared DeepSEA and our basic CNN+MLP for this purpose.

4.1 Which subset of sample pairs with sequence–profile disparity

Similar to our hypothesis tests in Section S2, we randomly drew 1% of the test samples, chose all pairs but those with zero interaction frequencies, and calculated their sequence similarity, (actual) label/profile similarity, and predicted label similarity (1 less the difference in predicted probability, averaged over all 919 epigenetic events / labels). We used sequence–profile disparity margin $\delta = 20\%$ (so separation is $2\delta = 40\%$, see Figure S1) to partition sampled pairs into three roughly equal-sized subsets. And we compared performances of DeepSEA and our CNN+MLP in the three subsets, using p-values (from one-sided Wilcoxon rank-sum tests) and Jensen–Shannon (JS) distances. Tables S3, S4, and S5 show that improvements were made among all 3 cell lines and all 3 subsets (low sequence high label similarity or "under", consistent sequence and label similarity,

and high sequence low label similarity or "over"). The largest improvement was for the "under" subset, indicating that long range interactions in chromatin 3D structure data are picked up in our models to boost prediction similarity for sequentially far yet structurally close DNA sequences.

Table S3: CNN+MLP using GM12878 chromatin structure versus DeepSEA

	low seq high label	consistent	high seq low label
p-value	3.62E-19±6.14E-19	8.10E-10±0.16E-10	4.74E-9±6.65E-9
JS distance	8.81E-3±6.18E-4	3.15E-3±2.06E-4	4.30E-3±6.18E-5

Table S4: CNN+MLP using IMR90 chromatin structure versus DeepSEA

	low seq high label	consistent	high seq low label
p-value	3.03E-24±6.01E-24	4.37E-13±8.75E-13	2.30E-9±4.59E-9
JS distance	8.77E-3±2.63E-4	3.35E-3±2.39E-4	4.63E-3±3.64E-4

Table S5: CNN+MLP using K562 chromatin structure versus DeepSEA

	low seq high label	consistent	high seq low label
p-value	6.38E-24±9.71E-24	4.22E-16±8.24E-16	1.32E-9±2.640E-9
JS distance	9.73E-3±7.61e-4	3.58E-3±1.27E-4	4.75E-3±2.07E-4

4.2 Which subset of epigenetic events: cell lines and event types

We did head-to-head comparison between DeepSEA and our CNN+MLP in AUROC or AUPRC and showed the scatter plots in Figure S3 where each symbol corresponded to one of the 919 epigenetic events to predict. We differentiated the symbols based on cell lines and event types and asked which subset of epigenetic events benefited more thanks to the introduction of input chromatin structure data. Results indicated that epigenetic events of the same cell line as that of the input chromatin structure and epigenetic events of transcription factor binding were among the biggest beneficiaries.

We also checked which type of epigenetic event prediction benefited more from the inclusion of 3D chromatin structure information. Table S6 showed that whereas chromatin accessibility benefited the most (which was intuitively satisfactory), histone marks did the least (albeit still the majority).

Table S6: Compared to DeepSEA, our CNN+MLP using additional chromatin structure information had its improvements split among chromatin accessibility, TF binding and histone marks. Among our 5 replicates, the one with median validation loss was used.

Epigenetic event subsets	Percentages of the epigenetic events with improved prediction, in terms of AUPRC
Chromatin accessibility	100% / 100% / 99.2 %
TF Binding	96.4% / 95% / 92.6 %
Histone marks	61.9% / 81.5% / 54.4 %

4.3 Chromatin 3D structure versus chromatin 1D order and accessibility

To further trace the origin of improvements, we compared our models using the input of chromatin 3D structure (graphs of chromatin interaction frequencies) versus ablated ones using the input

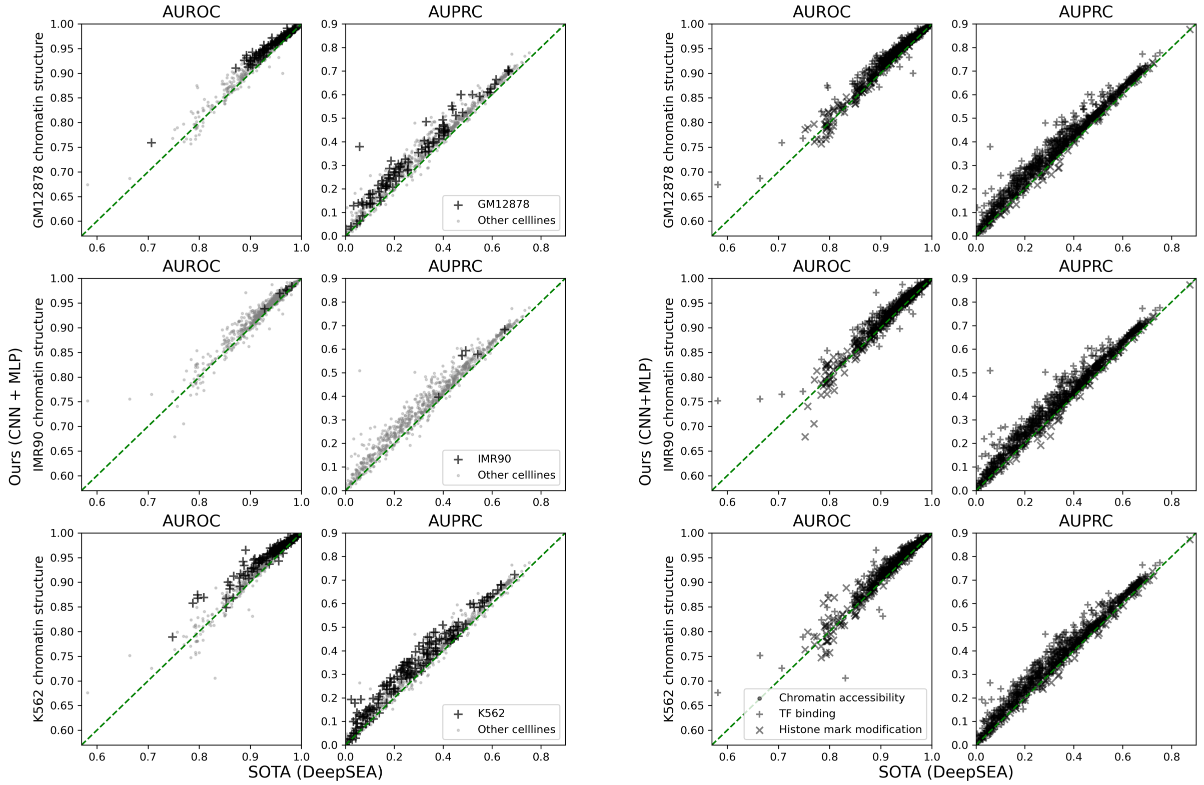

Figure S3: (Left) Using CNN+MLP to incorporate additional input of chromatin structure, epigentic profile prediction improved more against DeepSEA for epigenetic events associated with the same cell line as that of the input chromatin structure. (Right) The most extreme improvements were often for epigenetic events related to transcription factor binding.

of chromatin 1D structure (sequential graphs and chromatin accessibility). In addition, we also checked model variants where chromatin accessibility appends chromatin structure (where our normalization removed certain information). These models were described in Sec. 3.2. All models' local DNA sequence embedding was based on CNN as in DeepSEA.

Table S7 shows that when the global chromatin information is embedded with MLP, there was no significant difference between using chromatin accessibility versus using chromatin structure. However, when chromatin structure is encoded with GCN that is more compatible with graph-structured data, GCN (topology only) was much better than MLP (chromatin accessibility) by 1 to 2 standard deviations, which indicates that our models benefited from Hi-C the chromatin 3D structure information beyond the chromatin 1D accessibility. If the graphs were replaced by sequential ones confined in individual chromosomes, the predictive performances were drastically deteriorated (although still better in AUPRC than using no global chromatin embedding). Lastly, combining our most advanced chromatin embedding, GCN (sequence + topology), and MLP (chromatin accessibility MLP) could still improve AUPRC by 0.001. We believe that this is related to the fact that our normalization procedure for the interaction frequency matrices lost certain infor-

mation provided partially in chromatin accessibility (total counts).

Table S7: Comparing the accuracy of epigenetic profile prediction using chromatin 3D structures (as in interaction frequency graphs) versus using 1D orders (as in sequential graphs in individual chromosomes) and 1D accessibility (as in normalized total reads) as well as using 3D structures and 1D accessibility together. Local DNA sequence embedding is always available through CNN as in DeepSEA.

Chromatin Embedding	AUROC
None	0.933
MLP(unnormalized IF only)	0.934 ± 0.001 / 0.933 ± 0.001 / 0.934 ± 0.001
MLP(normalized IF only)	0.941 ± 0.001 / 0.940 ± 0.000 / 0.939 ± 0.002
MLP(chromatin accessibility only)	0.940 ± 0.001 / 0.938 ± 0.001 / 0.940 ± 0.001
MLP(normalized IF and chromatin accessibility)	0.941 ± 0.001 / 0.939 ± 0.004 / 0.370 ± 0.002
MLP(normalized IF) + MLP(chromatin accessibility) (default: 128-d)	0.940 ± 0.001 / 0.939 ± 0.001 / 0.940 ± 0.001
MLP(normalized IF) + MLP(chromatin accessibility) (64-d)	0.940 ± 0.000 / 0.939 ± 0.001 / 0.941 ± 0.000
GCN(sequential topology + sequence)	0.934 ± 0.001
GCN(topology only)	0.940 ± 0.001 / 0.940 ± 0.001 / 0.941 ± 0.000
GCN(topology + sequence)	0.942 ± 0.000 / 0.942 ± 0.000 / 0.943 ± 0.001
GCN(topology + sequence) + MLP(chromatin accessibility)	0.943 ± 0.000 / 0.943 ± 0.000 / 0.943 ± 0.000

Chromatin embedding	AUPRC
None	0.342
MLP(unnormalized IF only)	0.364 ± 0.002 / 0.355 ± 0.002 / 0.354 ± 0.005
MLP(normalized IF only)	0.375 ± 0.004 / 0.373 ± 0.003 / 0.370 ± 0.006
MLP(chromatin accessibility only)	0.374 ± 0.003 / 0.370 ± 0.002 / 0.373 ± 0.003
MLP(normalized IF and chromatin accessibility)	0.374 ± 0.003 / 0.369 ± 0.004 / 0.370 ± 0.002
MLP(normalized IF) + MLP(chromatin accessibility) (default: 128-d)	0.375 ± 0.000 / 0.371 ± 0.002 / 0.375 ± 0.003
MLP(normalized IF) + MLP(chromatin accessibility) (64-d)	0.374 ± 0.002 / 0.371 ± 0.002 / 0.375 ± 0.001
GCN(sequential topology + sequence)	0.355 ± 0.003
GCN (topology only)	0.379 ± 0.002 / 0.374 ± 0.003 / 0.377 ± 0.002
GCN(topology + sequence)	0.380 ± 0.001 / 0.379 ± 0.002 / 0.382 ± 0.002
GCN(topology + sequence) + MLP(chromatin accessibility)	0.381 ± 0.001 / 0.381 ± 0.002 / 0.383 ± 0.001

5 Sensitivity Analysis of Model Performances

We tested how sensitive our models are to input data, model architecture, model training, and data split, using CNN+MLP as an example. We also tested how sensitive our most advanced CNN+GCN (topology+sequence) is to the 100K-bp sequence embedding.

5.1 Impact of model input: resolution of chromatin structure Hi-C data

To analyze the impact of the input chromatin structure's resolution, we used same cell lines' data of lower resolutions (500K-bp and 1M-bp) to train the model and only changed the dimension of the input layer for chromatin structure embedding accordingly. As shown in Figure S4, model performances were relatively stable with regard to input chromatin structure's resolution and consistently above DeepSEA. Higher resolution of 100K-bp did perform better and even higher resolutions could help even more.

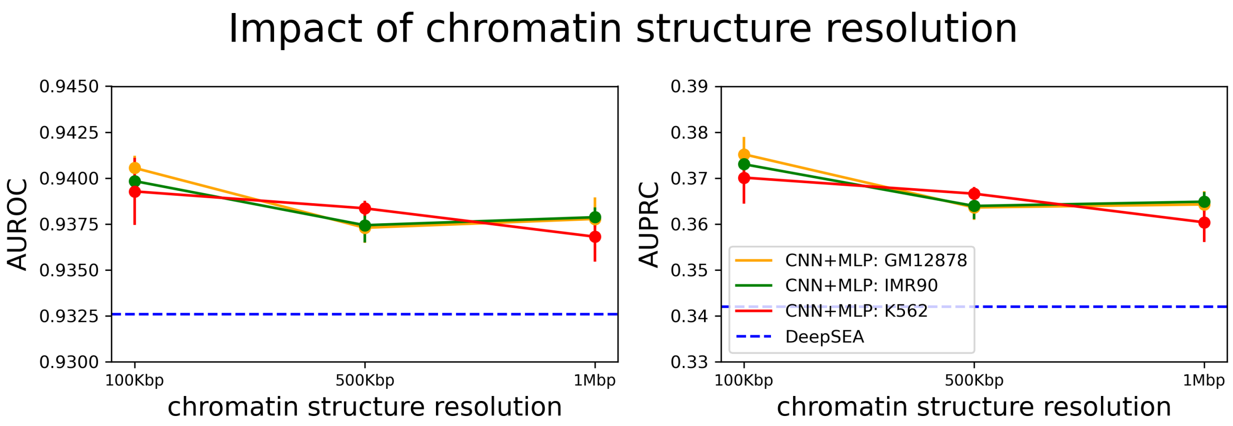

Figure S4: Epigenetic prediction performances of our CNN+MLP while using the input of different cell lines' chromatin structures of different resolutions. They all outperformed sequence-only DeepSEA (dashed line) and higher resolution (100K-bp) helped.

5.2 Impact of model architecture: dimension of chromatin embedding

With input chromatin structure's resolution fixed at 100K-bp, we tested on the dimension of the latent space embedding the input structure. We varied the output dimension of the last fully connected layer of MLP for chromatin embedding to 64, 128 (default), 256, and 512 and kept using the previously determined optimal hyperparameters. Figure S5 shows relative stable performances consistently outperforming DeepSEA, regardless of the embedding dimension. Reducing the latent dimension of chromatin structure embedding from the default 128 to 64 noticeably lowered the performances whereas increasing it to 256 or 512 did not help either. The results show a tricky balance between sequence embedding and structure embedding especially when DNA sequences and chromatin structures are of different resolutions.

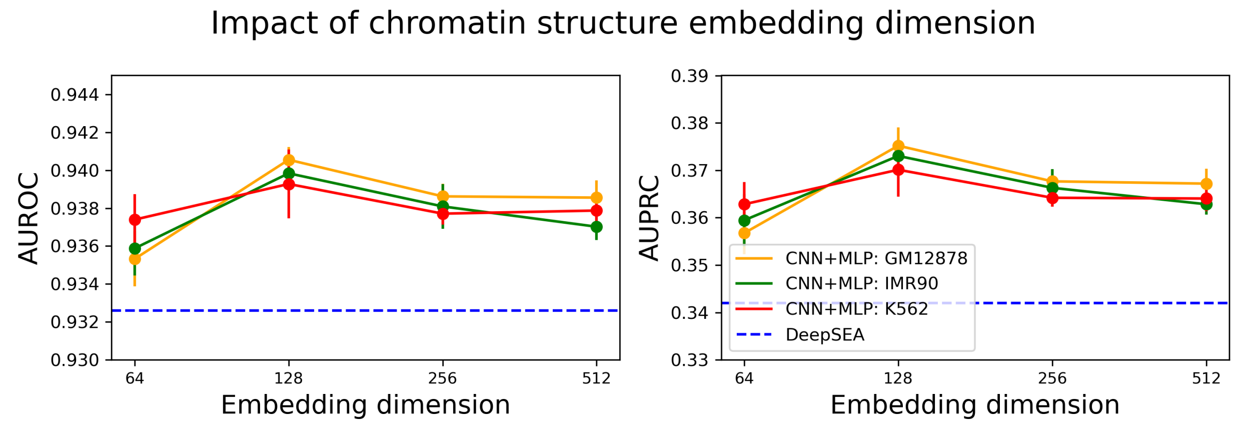

Figure S5: Epigenetic prediction performances of our CNN+MLP while using the input of different cell lines' chromatin structures embedded in different dimensions. They all outperformed sequence-only DeepSEA (dashed line).

5.3 Impact of model training: parameter initialization and normalization

We have tested the impact of changing the parameter initialization or adopting batch normalization, without changing the model architecture. In our default setting, the parameters of the fully connected layers in the chromatin embedding and after the concatenation of embeddings were initialized following the uniform distribution $U(-\frac{1}{\sqrt{k}}, \frac{1}{\sqrt{k}})$, where k is the number of the features. Here we have additionally tested the Xavier uniform initialization with the gain parameter (for scaling) at 1 as well as the Xavier normal initialization with gain at 1.0, which are implemented in PyTorch. We also tested adding batch normalization for all fully connected layers for chromatin structure embedding (right branch in Figure 1 of the main text). We used the same L1 and L2 regularization weights and the same learning rate, as the optimal values in the default CNN+MLP, for the tests.

Table S8: Our CNN+MLP models' AUROC using different initializers or using batch normalization.

	GM12878	IMR90	K562
default initialization	0.941±0.001	0.940±0.000	0.939±0.002
xavier uniform initialization	0.939±0.001	0.938±0.001	0.939±0.001
xavier normal initialization	0.941±0.001	0.939±0.001	0.939±0.001
batch normalization	0.940±0.001	0.939±0.002	0.939±0.001

Table S9: Our CNN+MLP models' AUPRC using different initializers or using batch normalization.

	GM12878	IMR90	K562
default initialization	0.375±0.004	0.373±0.003	0.370±0.005
xavier uniform initialization	0.372±0.003	0.369±0.003	0.370±0.005
xavier normal initialization	0.371±0.004	0.370±0.002	0.371±0.003
batch normalization	0.373±0.003	0.371±0.003	0.373±0.0045

As shown in the two tables above, adopting a different initializer or adding batch normalization did not significantly change the performance for our CNN+MLP models. We thus adopted the initializer and no batch normalization while training all our models.

5.4 Impact of data splitting: completely removing chromosome overlaps across data splits.

Data splitting strategies are important to assessing machine learning performances. Although there were no samples (kilobase regions) shared among training, validation, or testing sets, our data split following DeepSEA did have Chromosome 7 present in both training and validation sets as part of the training set and the entire validation set were sampled (without an overlap in kilobase regions) from Chromosome 7.

To completely remove overlaps in chromosomes, we retained all samples in Chromosome 7, 8, and 9 in the training set, and randomly split the nineteen remaining autosomes into two (testing set), one (validation set), and sixteen (to append Chromosome 7–9 for training set). Such random split was repeated six times and for each data split we re-trained DeepSEA (sequence only) and our basic CNN + MLP (sequence + structure) five times each for mean and standard deviations in AUROC and AUPRC. Tables S10 and S11 show that the improvements due to the inclusion of chromatin structure data were consistent across various splitting scenarios and were even more pronounced compared to those observed previously in the DeepSEA data splits.

Table S10: The improvement in epigenetic prediction (AUROC) due to the additional chromatin structure data was insensitive to data splitting.

Val/Test chromosomes	DeepSEA	Our CNN+MLP GM12878/IMR90/K562
chr12/chr2, chr14	0.931±0.000	0.946±0.000/0.947±0.000/0.945±0.000
chr20/chr18, chr22	0.919±0.001	0.933±0.001/0.932±0.001/0.926±0.000
chr3/chr5, chr10	0.927±0.000	0.943±0.000/0.941±0.001/0.939±0.000
chr13/chr6, chr17	0.926±0.000	0.943±0.000/0.942±0.001/0.939±0.000
chr15/chr11, chr21	0.924±0.000	0.939±0.000/0.939±0.000/0.936±0.000
chr19/chr1, chr4	0.926±0.001	0.941±0.000/0.942±0.001/0.941±0.000

Table S11: The improvement in epigenetic prediction (AUPRC) due to the additional chromatin structure data was insensitive to data splitting.

Val/Test chromosomes	DeepSEA	Our CNN+MLP GM12878/IMR90/K562
chr12/chr2, chr14	0.347±0.001	0.412±0.002/0.415±0.001/0.404±0.002
chr20/chr18, chr22	0.331±0.001	0.382±0.001/0.379±0.003/0.369±0.003
chr3/chr5, chr10	0.338±0.001	0.406±0.001/0.391±0.002/0.387±0.001
chr13/chr6, chr17	0.351±0.001	0.421±0.002/0.414±0.002/0.406±0.001
chr15/chr11, chr21	0.350±0.001	0.408±0.001/0.406±0.001/0.395±0.001
chr19/chr1, chr4	0.338±0.001	0.395±0.001/0.401±0.003/0.403±0.001

5.5 When training and testing Hi-C data are from different cell lines

Please see the comparisons in Figure S6 (next page).

5.6 Impact of 100K-bp sequence embedding to CNN+GCN(topology+sequence): DNABERT vs. DeepSEA

Considering that language model-based DNABERT may contain more contextual information, we replaced it with DNABERT (second last layer's 925-dimensional output as in the published version) to embed the 100K-bp regions in our advanced CNN+GCN(topology + sequence). Table S12 shows that our model was still able to benefit from DeepSEA-based embedding of the 100K-bp sequences (nodes) where AUPRC were 0.001–0.003 higher than GCN (topology only). It is slightly worse than, equal to, and slight better than that of DNABERT with equal frequencies (two out of six combinations of AUROC/AUPRC and cell lines).

Table S12: AUROC and AUPRC in epigenetic profile prediction for CNN + GNN (topology + sequence) where the 100K-bp node embedding in GNN was based on DNABERT versus DeepSEA.

Method	AUROC
GCN (topology only)	0.940 ± 0.001 / 0.940 ± 0.000 / 0.941 ± 0.000
GCN (topology + sequence (DNABERT))	0.941 ± 0.001 / 0.942 ± 0.000 / 0.941 ± 0.001
GCN (topology + sequence (DeepSEA))	0.942 ± 0.001 / 0.942 ± 0.000 / 0.942 ± 0.000

Method	AUPRC
GCN (topology only)	0.379 ± 0.002 / 0.374 ± 0.003 / 0.377 ± 0.002
GCN (topology + sequence (DNABERT))	0.380 ± 0.001 / 0.379 ± 0.002 / 0.382 ± 0.002
GCN (topology + sequence (DeepSEA))	0.380 ± 0.002 / 0.377 ± 0.002 / 0.379 ± 0.002

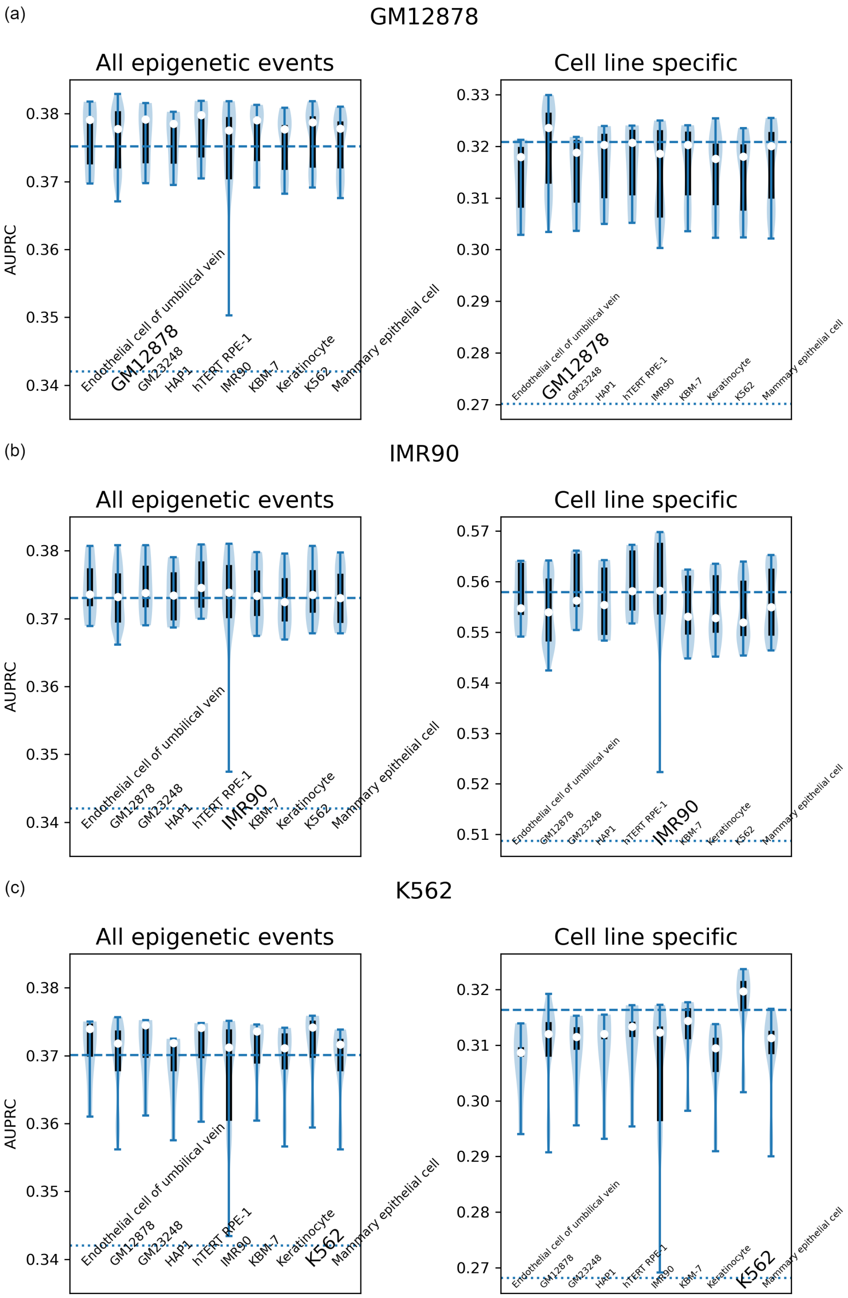

Figure S6: Violin plots of our CNN+MLP's performance when it is trained using Hi-C chromatin structure data for cell lines (a) GM12878, (b) IMR90, and (c) K562, respectively, but tested with other cell lines' Hi-C chromatin structure data (with corresponding name below each violin plot). Plotted are AUPRC distributions over five trials times (left) all epigenetic events or (right) those related to cell lines (a) GM12878, (b) IMR90, and (c) K562. The white dot, the box, and the whiskers indicate the median, the 25%–75% percentile, and the extremes, respectively. The dashed line represents the mean performance when each model is tested with the matched cell line's Hi-C data. The dotted line below represents the performance of sequence-only DeepSEA.

6 Extracting transcription factor (TF) binding motifs from learned epigenetic predictors

6.1 Overall comparison across models

We report the numbers of convolutional kernels (out of 320, in the first layer of various models) that were found to match one or more known transcription factor binding motifs in Table S13. We also show the numbers of matched motifs in Venn diagrams in FigureS7. Note that our local DNA sequence embedding was based on CNN from DeepSEA or CNN/RNN from DanQ, where kernel sizes were 8 and 26, respectively. Thus directly comparing DeepSEA and DanQ (under the same E value cutoff in TOMTOM) would be unfair; and our comparisons are focused on either version without or with chromatin structure input (under various cell lines). Clearly with chromatin structure as input, our models showed complementarity to sequence-only models DeepSEA or DanQ.

Table S13: Number of kernels similar to known TF binding motifs.

	GM12878	IMR90	K562
DeepSEA (kernel size 8)		23	
CNN (kerel size 8) + MLP	25	24	25
CNN+GCN (all 1 node feat.)	27	28	25
CNN+GCN (DNABERT node feat.)	24	27	26
DanQ (kernel size 26)		169	
CNN (kernel size 26) /RNN + MLP	167	164	166
CNN/RNN+GCN (all 1 node feat.)	167	166	166
CNN/RNN+GCN (DNABERT node feat.)	165	168	168

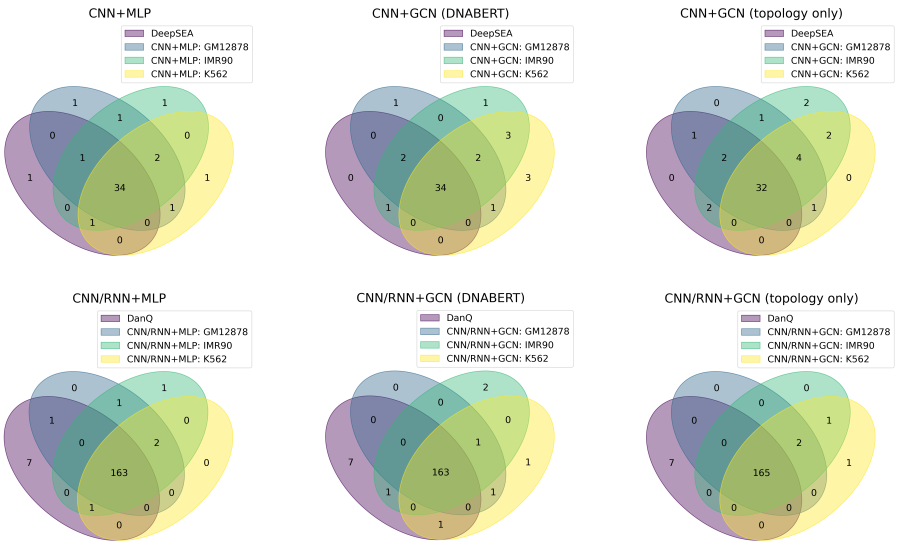

Figure S7: The numbers of known transcription factor binding motifs that were found a match to the sequence profiles in various models' learned convolutional kernels.

In the following subsections we analyze the specific motifs uniquely identified or missed by our

models compared to DeepSEA or DanQ. We did not analyze our models when DNABERT was used because in such a case DNA sequence embedding occurs in both DNA sequence-embedding CNN and chromatin-embedding GCN and it would be unreasonable to only examine the first convolutional layer of CNN to extract motifs.

6.2 Using CNN of kernel size 8 as in DeepSEA

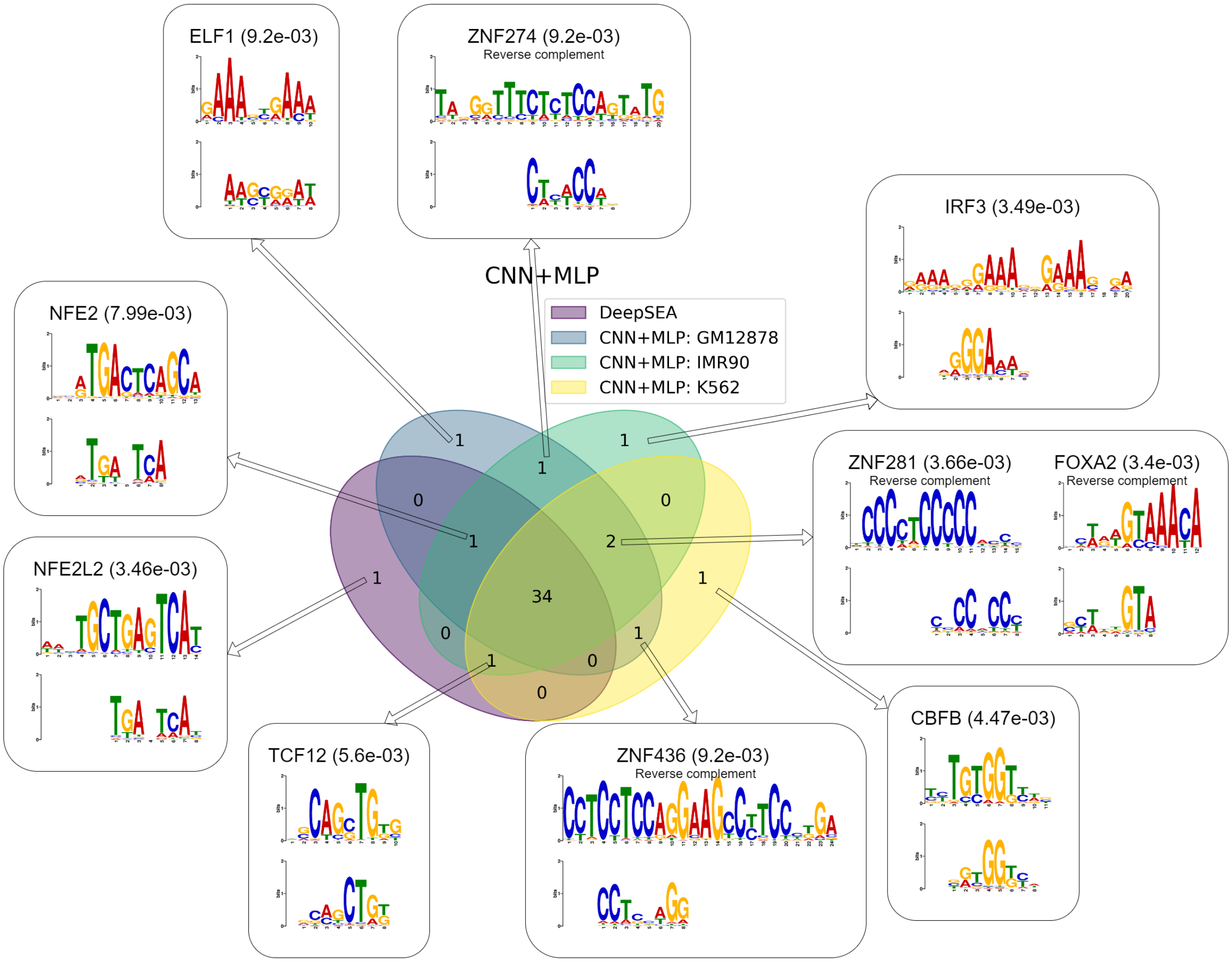

Figure S8: The numbers of motifs identified by CNN+MLP (using various cell lines' chromatin structures) versus DeepSEA.

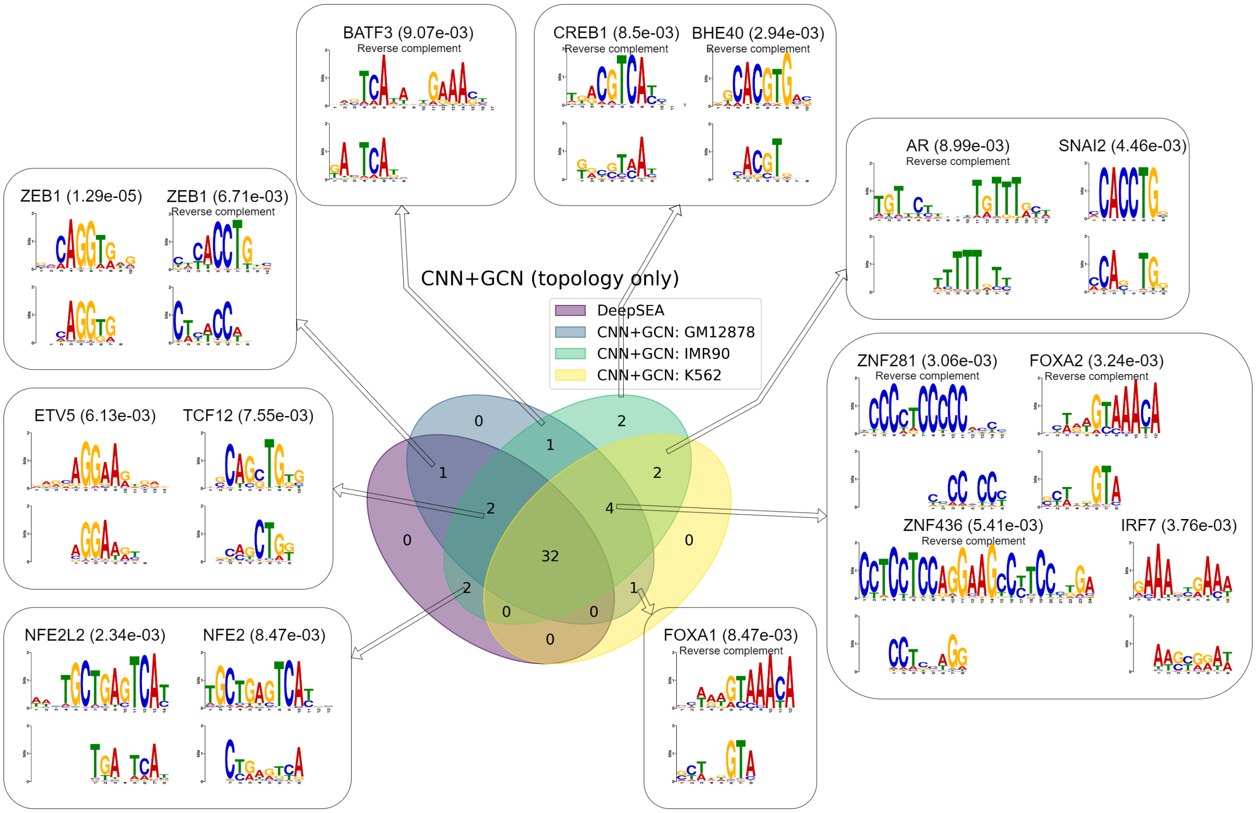

Figure S9: The numbers of motifs identified by CNN+GCN (topology only) (using various cell lines' chromatin structures) versus DeepSEA.

6.3 Motif extraction using CNN of kernel size 26 as in DanQ

Our CNN/RNN+GCN consistently identified two motifs, TFs for genes POU3F1 (both strands) and TFDP1, that were missed by DanQ. The discovery of these two motifs was attributed to the use of chromatin structure that suggests long-range interactions. Specifically, POU3F1 (on Chromosome 1 or Chr. 1 in short) is a transcriptional activator that binds cooperatively with SOX4 (on Chromosome 6), SOX11 (Chr. 2) or SOX12 (Chr. 20). According to the chromatin topology defined by any of the 3 cell lines' interaction frequency matrix, POU3F1's region (100kbp resolution) is directly connected or separated by a common neighbor to SOX4, SOX11 and SOX12 regions. Without chromatin structures, the cross-chromosome TF cooperation would be much harder to identify from sequence alone. Similarly, TFDP1 (Chr. 13) encodes a transcription factor that functions cooperatively with E2F family members through the E2 recognition sites and plays a critical role in cell cycle regulation. According to the chromatin structure topology defined in the input Hi-C data, regardless of the cell line, the pseudo genes of TFDP1P1 (Chr. 1), TFDP1P2 (Chr. X), and TFDP1P3 (Chr. 15), as well as other genes important in cell cycle regulation including CCNA1 (Chr. 13), CCNA2 (Chr. 4), CCND1 (Chr. 11), CCND2 (on Chr. 12), CDK2 (Chr. 12), MYB (Chr. 6), E2F1 (Chr. 20), E2F2 (Chr. 1), E2F3 (Chr. 20) and CDC25A (Chr. 3) are all directly connected to or share a common neighbor with the TFDP1 region (100K-bp).

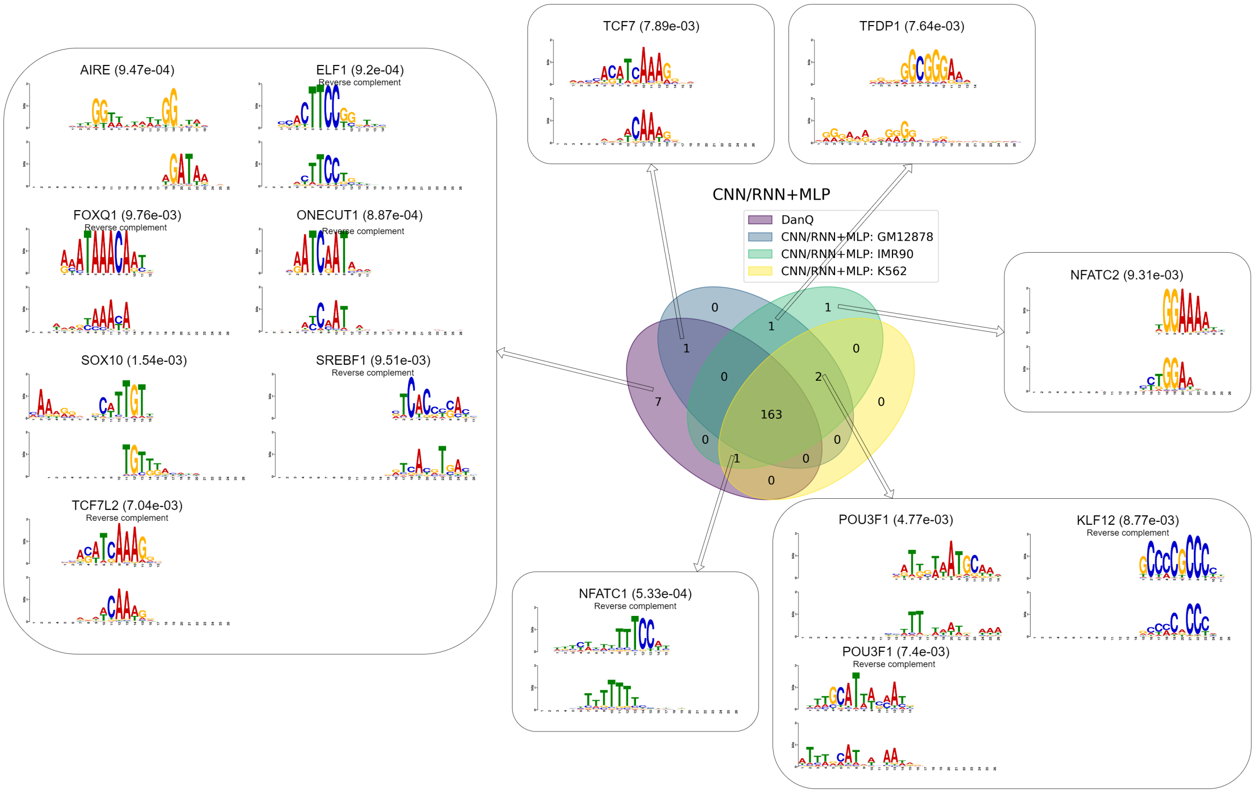

Figure S10: The numbers of motifs identified by CNN/RNN+MLP (using various cell lines' chromatin structures) versus DanQ.

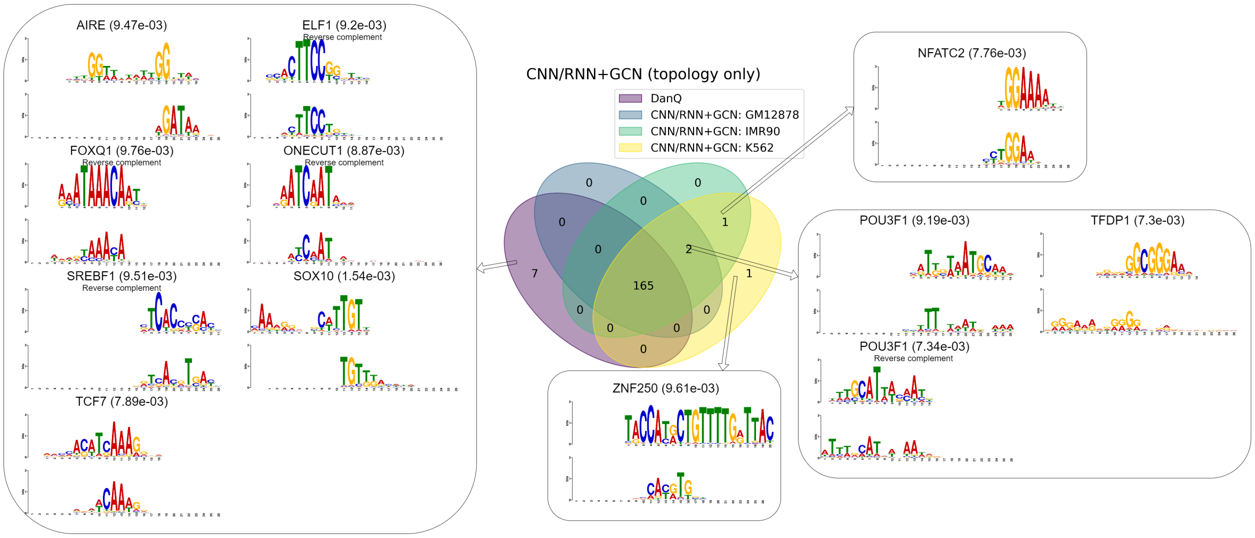

Figure S11: The numbers of motifs identified by CNN/RNN+GCN (topology only) (using various cell lines' chromatin structures) versus DanQ

6.4 Understanding the false negatives

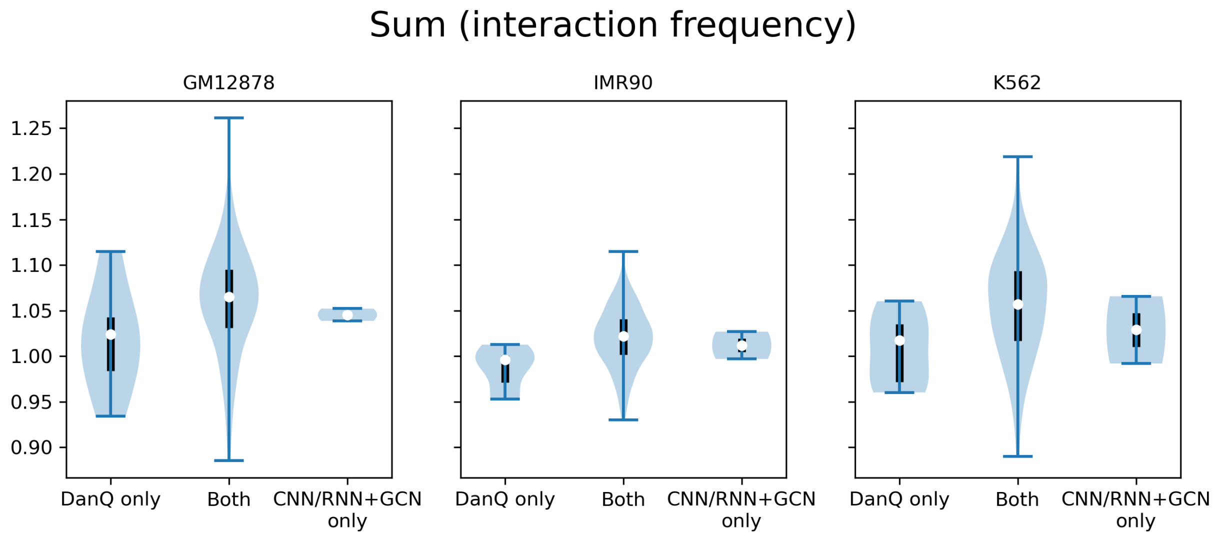

Figure S12: To have a better understanding of the motifs found by sequence-only DanQ but missed by our CNN/RNN+GCN topology only, we compared the sums of normalized interaction frequencies of the uniquely missed 7 genes, the commonly found genes, and the uniquely identified genes of our model.

7 Predicting noncoding variant effect: eQTL

7.1 Performances

We first examined the distribution of the eQTLs across chromosomes. As shown in Figure S13, the amounts of eQTLs per chromosome length were of small variances except for chromosomes 17 and 19.

We next held out eQTLs from chromosome 8 and 9 for hyperparameter tuning. The regularization weights resulting in the highest 5-fold cross validation AUROC for the held-out set were selected and reported in Table S14. We then randomly split the rest chromosomes into 5 folds for 5-fold cross validation (with hyperparameter already tuned), while having similar amounts of chromosomes across folds: {chr2, chr3, chr7, chr14, chr16}, {chr4, chr10, chr12, chr19}, {chr1, chr13, chr17, chrX, chrY}, {chr5, chr15, chr18, chr20} and {chr6, chr11, chr21, chr22}. To make sure that chrX and chrY are in the same fold, those two chromosomes were essentially treated one when chromsomes were split into 5 folds.

Table S14: L2 regularization term used in 5-fold cross validation

	DeepSEA	CNN+MLP	CNN+GCNw/DNABERT	DanQ	CNN/RNN+MLP	CNN/RNN+GCNw/DNABERT
GM12878	1E1	1E-2	1E-3	1E-1	1E-2	1E-2
IMR90	1E0	1E-2	1E-2	1E-1	1E-2	1E-2

We used the cell line-specific epigenetic predictions from our models to predict the eQTL's effect (expression level increasing or decreasing). Besides the performances of our basic CNN+MLP reported in the main text, we also report here the performances of CNN+GCN with DNABERT, CNN/RNN+MLP, and CNN/RNN+GCN with DNABERT.

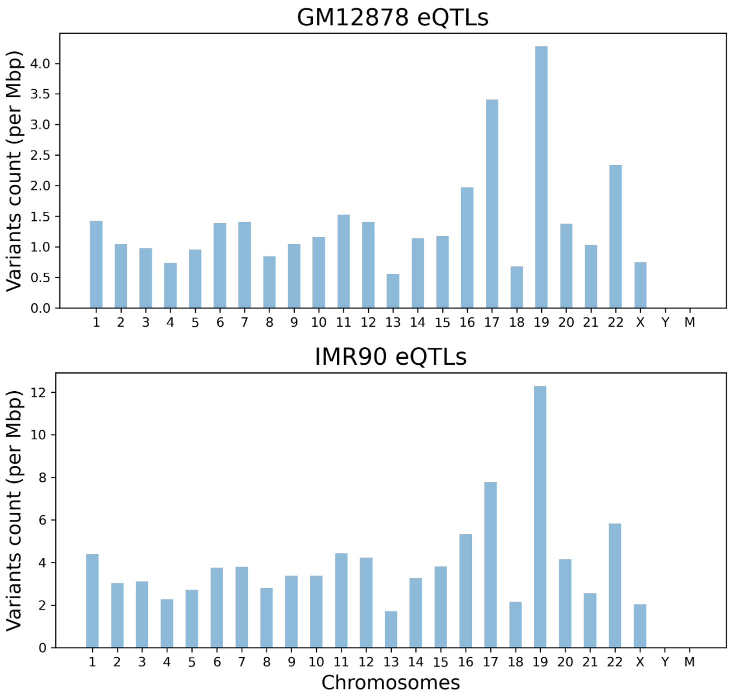

Figure S13: The distribution of the eQTLs across chromosomes.

As shown below, our chromatin structure-informed various models could outperform the corresponding sequence only model (DeepSEA or DanQ) in eQTL effect prediction, especially when the effects were strong.

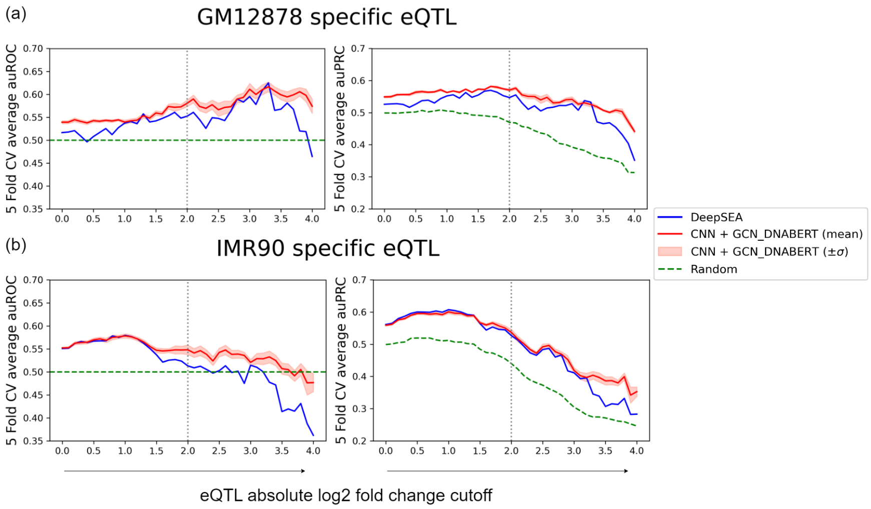

Figure S14: Using cell line specific epigenetic predictions from our CNN+GCN (with DNABERT to embed chromatin sequence and structure together) or DeepSEA to predict eQTL effect. For eQTLs with strong effect (the cutoff of expression log2 fold change at 2), our model for cell line GM12878 was 3.95σ better (in AUROC) and 5.91σ better (in AUPRC) than DeepSEA; and that for cell line IMR90 was 3.39σ better (in AUROC) and 1.03σ better (in AUPRC) than DeepSEA. For the stronger effect eQTLs (expression log2 fold change cutoff at 2.5), our model for cell line GM12878 was 1.31σ better (in AUROC) and 3.25σ better (in AUPRC) than DeepSEA; and that for cell line IMR90 was 4.28σ better (in AUROC) and 0.99σ better (in AUPRC) than DeepSEA.

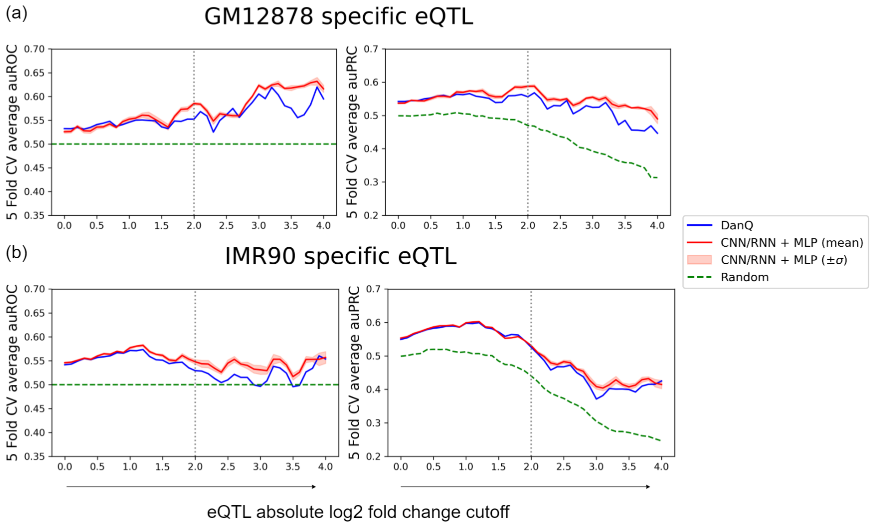

Figure S15: Using cell line specific epigenetic predictions from our CNN/RNN+MLP or DanQ to predict eQTL effect. For eQTLs with strong effect (the cutoff of expression log2 fold change at 2), our model for cell line GM12878 was 12.32σ better (in AUROC) and 9.67σ better (in AUPRC) than DanQ; and that for cell line IMR90 was 4.15σ better (in AUROC) and 3.95σ better (in AUPRC) than DanQ. For the stronger effect eQTLs (expression log2 fold change cutoff at 2.5), our model for cell line GM12878 was 0.03σ better (in AUROC) and 4.26σ better (in AUPRC) than DanQ; and that for cell line IMR90 was 5.14σ better (in AUROC) and 3.36σ better (in AUPRC) than DanQ.

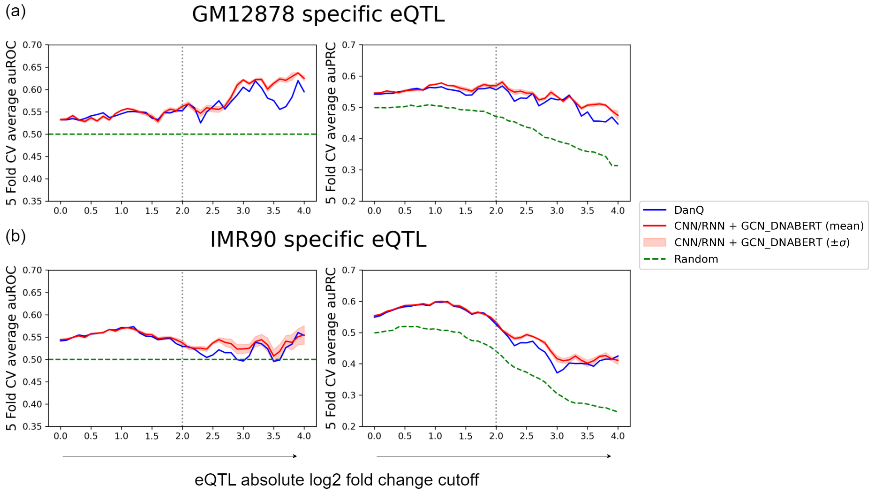

Figure S16: Using cell line specific epigenetic predictions from our CNN/RNN+GCN (with DNABERT to embed chromatin sequence and structure together) or DanQ to predict eQTL effect. For eQTLs with strong effect (the cutoff of expression log2 fold change at 2), our model for cell line GM12878 was 2.11σ better (in AUROC) and 2.51σ better (in AUPRC) than the DanQ; and that for cell line IMR90 was 1.38σ better (in AUROC) and 1.53σ better (in AUPRC) than DanQ. For the stronger effect eQTLs (expression log2 fold change cutoff at 2.5), our model for cell line GM12878 was 0.62σ worse (in auROC) and 2.33σ better (in AUPRC) than DeepSEA; and that for cell line IMR90 was 10.08σ better (in AUROC) and 6.91σ better (in AUPRC) than DanQ.

7.2 Impact of epigenetic events as features

Our eQTL predictors used epigenetic profile predictions as features. Specifically, these features corresponded to 919 epigenetic events curated by DeepSEA. Meanwhile, the number of epigenetic events has been increasing over years as epigenetics data grow: 2,002 in Expecto [6] and 21,907 in Sei [5]. Whereas more epigenetic features are expected to be more predictive features for eQTL prediction, we examined the impact explicitly by reducing the (919) epigenetic events that we had used.

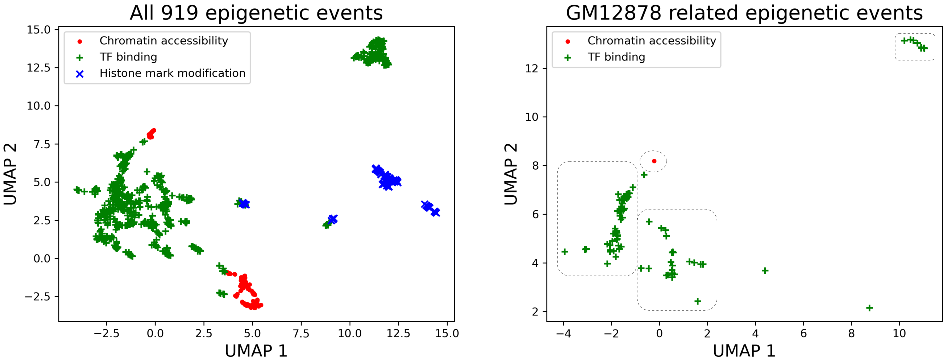

Figure S17: Clustering visualization of (Left) all 919 epigenetic events and (Right) the subset of 91 GM12878 related epigenetic events.

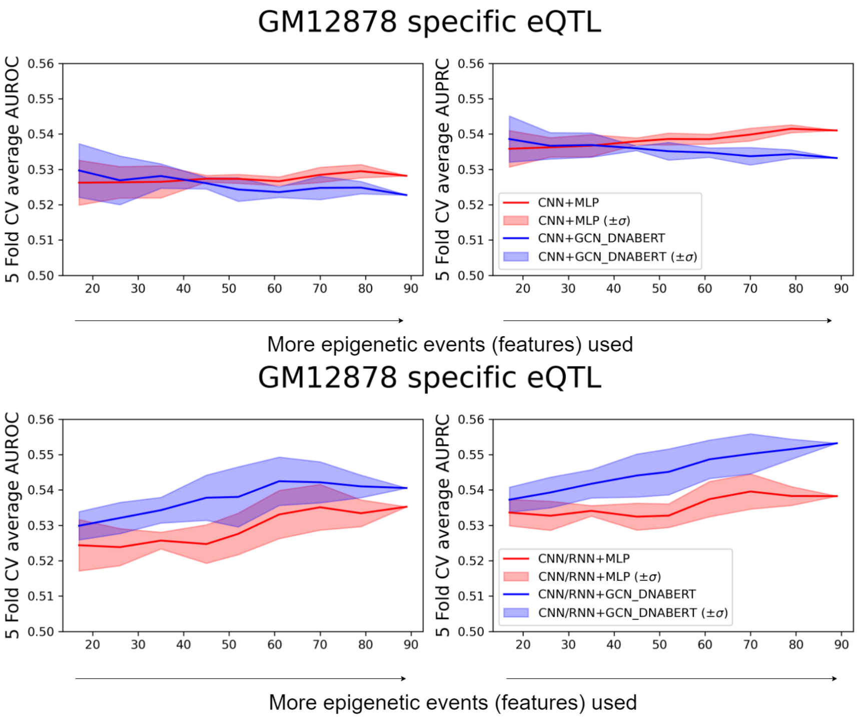

Figure S18: From left to right, we are using more and more GM12878 related epigenetic events and reached higher AUROC (left) and AUPRC (right).

Table S15: Using more epigenetic events (features) resulted in higher AUROC and AUPRC for GM12878 eQTL prediction.

	CNN+MLP	CNN+GCN(DNABERT)	CNN/RNN+MLP	CNN/RNN+GCN(DNABERT)
AUROC (20% features)	0.526±0.006	0.530±0.008	0.524±0.007	0.530±0.004
AUROC (100% features)	0.528	0.523	0.535	0.541
AUPRC (20% features)	0.536±0.005	0.539±0.007	0.534±0.004	0.537±0.004
AUPRC (100% features)	0.541	0.533	0.538	0.553

8 Predicting noncoding variant effect: Pathogenicity

8.1 Data curation

The splice-cite variants occur at the boundary between an intron and an exon, which may disrupt RNA splicing and result in protein-coding sequence changes. We therefore removed the splicing variants whose variants effects cannot be adequately described by the epigenetic profile changes. With the help of VEP (variant effect predictor) [7], we removed all variants with the splicing related consequences (splice_acceptor_variant, splice_donor_variant, splice_region_variant, splice_donor_5th_base_variant, splice_donor_region_variant, splice_polypyrimidine_tract_variant). 455 (out of 721) pathogenic variants and 33 (out of 7226) benign variants have been removed from ncVarDB. To have a fair comparison, we also removed 1 pathogenic variant and 27 benign variants that were used as training samples by CADD. As a result, we had 265 pathogenic variants and 7167 benign variants, in total 7432 variants, before data splits.

8.2 Unsupervised "zero-shot" performances

We first report the unsupervised "zero-shot" learning performances on the test set curated from ncVarDB, by holding out 500 variants (400 for few shot learning, 100 for validation). The test set has 246 pathogenic variants and 6,686 pathogenic variants, in total 6932 variants with positive rate 3.7%. No pathogenic/benign variant data was used to re-train our models. Instead, absolute differences in epigenetic probabilities from pre-trained epigenetic predictors were used, in comparison to those from the sequence-only model. The results are summarized in Tables S16–S23.

Table S16: Unsupervised model prediction AUROC on ncVarDB: DeepSEA vs CNN+MLP.

	DeepSEA	Our GM12878	Our IMR90	Our K562
Chromatin accessibility	0.764	0.643 ± 0.006	0.735 ± 0.016	0.732 ± 0.005
TF binding	0.757	0.652 ± 0.010	0.733 ± 0.033	0.759 ± 0.004
Histone mark modification	0.774	0.733 ± 0.013	0.804 ± 0.005	0.802 ± 0.008
Weighted across 3 types	0.765	0.708 ± 0.011	0.790 ± 0.009	0.786 ± 0.005
All features	0.764	0.699 ± 0.011	0.782 ± 0.012	0.782 ± 0.004

Table S17: Unsupervised model prediction AUPRC on ncVarDB: DeepSEA vs CNN+MLP.

	DeepSEA	Our GM12878	Our IMR90	Our K562
Chromatin accessibility	0.098	0.092 ± 0.002	0.109 ± 0.002	0.107 ± 0.003
TF binding	0.148	0.141 ± 0.004	0.152 ± 0.006	0.163 ± 0.004
Histone mark modification	0.161	0.114 ± 0.007	0.163 ± 0.005	0.167 ± 0.018
Weighted across 3 types	0.128	0.116 ± 0.002	0.141 ± 0.002	0.141 ± 0.005
All features	0.142	0.134 ± 0.002	0.154 ± 0.003	0.159 ± 0.006

Table S18: Unsupervised model prediction AUROC on ncVarDB: DeepSEA vs CNN+GCN (DNABERT).

	DeepSEA	Our GM12878	Our IMR90	Our K562
Chromatin accessibility	0.710	0.763 ± 0.006	0.765 ± 0.011	0.760 ± 0.010
TF binding	0.757	0.805 ± 0.005	0.766 ± 0.013	0.816 ± 0.003
Histone mark modification	0.774	0.750 ± 0.005	0.747 ± 0.005	0.753 ± 0.005
Weighted across 3 types	0.765	0.778 ± 0.004	0.774 ± 0.005	0.780 ± 0.004
All features	0.764	0.794 ± 0.004	0.777 ± 0.006	0.801 ± 0.004

Table S19: Unsupervised model prediction AUPRC on ncVarDB: DeepSEA vs CNN+GCN (DNABERT).

	DeepSEA	Our GM12878	Our IMR90	Our K562
Chromatin accessibility	0.098	0.116 ± 0.004	0.125 ± 0.004	0.115 ± 0.005
TF binding	0.148	0.184 ± 0.003	0.156 ± 0.006	0.196 ± 0.005
Histone mark modification	0.161	0.114 ± 0.003	0.114 ± 0.006	0.108 ± 0.006
Weighted across 3 types	0.128	0.135 ± 0.004	0.140 ± 0.002	0.135 ± 0.004
All features	0.142	0.162 ± 0.004	0.155 ± 0.003	0.165 ± 0.005

Table S20: Unsupervised model prediction AUROC on ncVarDB: DanQ vs CNN/RNN+MLP.

	DanQ	Our GM12878	Our IMR90	Our K562
Chromatin accessibility	0.705	0.681 ± 0.007	0.731 ± 0.006	0.727 ± 0.003
TF binding	0.741	0.694 ± 0.010	0.751 ± 0.006	0.774 ± 0.001
Histone mark modification	0.757	0.768 ± 0.006	0.800 ± 0.006	0.795 ± 0.004
Weighted across 3 types	0.754	0.744 ± 0.008	0.786 ± 0.004	0.782 ± 0.003
All features	0.754	0.737 ± 0.009	0.783 ± 0.002	0.785 ± 0.003

Table S21: Unsupervised model prediction AUPRC on ncVarDB: DanQ vs CNN/RNN+MLP.

	DanQ	Our GM12878	Our IMR90	Our K562
Chromatin accessibility	0.097	0.090 ± 0.001	0.102 ± 0.002	0.097 ± 0.001
TF binding	0.134	0.138 ± 0.003	0.148 ± 0.003	0.161 ± 0.002
Histone mark modification	0.151	0.115 ± 0.004	0.148 ± 0.007	0.150 ± 0.005
Weighted across 3 types	0.129	0.116 ± 0.002	0.134 ± 0.003	0.133 ± 0.002
All features	0.135	0.133 ± 0.002	0.147 ± 0.002	0.151 ± 0.002

Table S22: Unsupervised models prediction AUROC on ncVarDB: DanQ vs CNN/RNN+GCN(DNABERT).

	DanQ	Our GM12878	Our IMR90	Our K562
Chromatin accessibility	0.705	0.786 ± 0.003	0.779 ± 0.003	0.777 ± 0.003
TF binding	0.741	0.811 ± 0.002	0.792 ± 0.004	0.811 ± 0.002
Histone mark modification	0.757	0.744 ± 0.004	0.723 ± 0.004	0.749 ± 0.008
Weighted across 3 types	0.754	0.790 ± 0.003	0.775 ± 0.002	0.785 ± 0.003
All features	0.754	0.804 ± 0.003	0.787 ± 0.003	0.802 ± 0.001

Table S23: Unsupervised models prediction AUPRC on ncVarDB: DanQ vs CNN/RNN+GCN(DNABERT).

	DanQ	Our GM12878	Our IMR90	Our K562
Chromatin accessibility	0.097	0.132 ± 0.004	0.132 ± 0.004	0.124 ± 0.003
TF binding	0.134	0.205 ± 0.006	0.171 ± 0.006	0.199 ± 0.001
Histone mark modification	0.151	0.110 ± 0.004	0.094 ± 0.004	0.105 ± 0.007
Weighted across 3 types	0.129	0.153 ± 0.004	0.144 ± 0.003	0.145 ± 0.002
All features	0.135	0.184 ± 0.006	0.164 ± 0.005	0.176 ± 0.002

8.3 Supervised "few-shot" performances

In the main text, we showed that our Siamese neural network with pretrained epigenetic encoders, either CNN/RNN+MLP or CNN/RNN+GCN (with DNABERT), consistently further improved pathogenicity classification against zero-shot learning, using as few as tens of pathogenicity-labeled variants.

Among the 6,932 testing variants, we have found 464 variants for which at least one of CADD, DANN, FATHMM-XF failed to make inference. After checking these SOTA models' variant effect predictions summarized by the ncVarDB [8], we found that CADD does not score for variants on chromosome M. At the same time, based on DANN's pre-computed scores, there is no score for chromosome Y and M. FATHMM-XF does not score for insertions, deletions and more than one nucleotide long substitutions. These 464 variants are split based on their types: 259 substitution, 154 deletion, and 51 insertion variants. They are also split based on the genomic position: 289 intronic, 11 intergenic, 97 ncRNA, 22 3'UTR and 45 5'UTR variants. A summary is in Figure S19.

Our models can make inference on the subset of test variants where SOTA models (such as

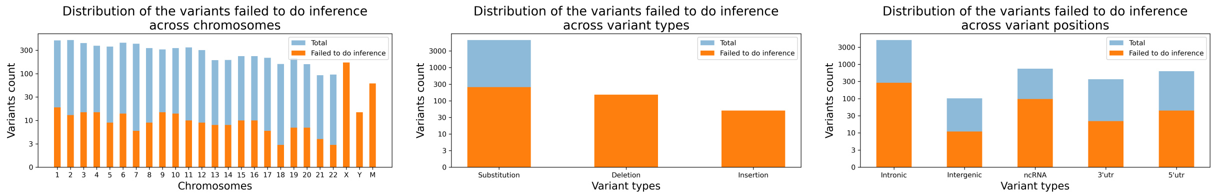

Figure S19: Summary of the 464 variants from ncVarDB that at least one of CADD, DANN, FATHMM-XF failed to make inference. Across all chromosomes, all variant types and variant positions, what proportion of the variants failed to receive inference is shown in the figure.

CADD, DANN, or FATHMM-XF) failed to make predictions. So, our models are more generally applicable regarding the variant types and positions. Clearly, as shown in Figure 7 of the main text for all test variants, the performances for the subset of test variants in Figure S20 also had the upward trend while using more few-shot training examples. AUPRC values for this subset were better than those for the overall test set.

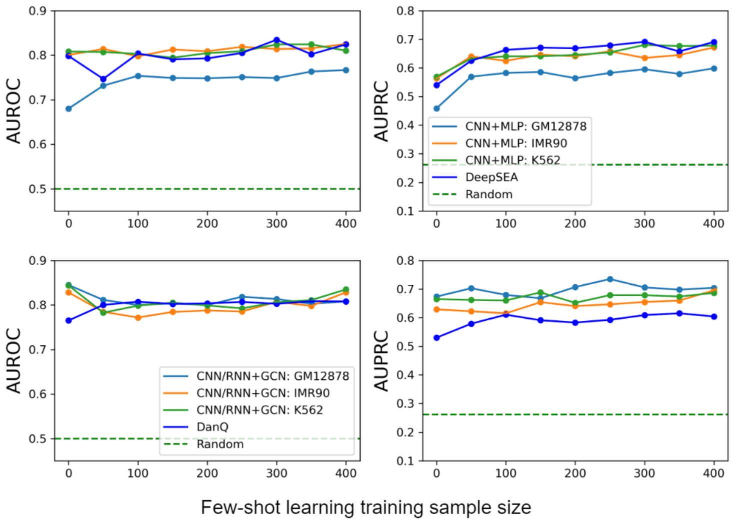

Figure S20: Our CNN+MLP (top) and CNN/RNN+GCN with DNABERT embedded node feature (bottom) prediction AUROC and AUPRC focusing on the subset that at least one of CADD, DANN, FATHMM-XF failed to make inference.

We also compared our proposed methods (few-shot using 400 training samples) with CADD, DANN, FATHMM-XF, as well as the PhyloP 100-way conservation score [9], on the subset of our test set where they can all make predictions, containing 153 pathogenic variants and 6315 benign variants with positive rate 2.4%. We also decomposed those variants into 5 subsets, in terms of the variant positions. We reported these results in Tables S24 (AUROC) and S25 (AUPRC).

Table S24: Comparing our proposed methods (400-shot) and the SOTA models on the subset of test set where all models can make predictions. AUROCs are reported

	All	Intronic	Intergenic	ncRNA	3'utr	5'utr
Conservation-based predictor						
PhyloP 100-way	0.678	0.559	0.927	0.848	0.777	0.702
Predictors trained on large training sets (up to tens of millions)						
CADD v1.4	0.856	0.792	0.982	0.920	0.894	0.800
DANN	0.762	0.631	0.978	0.868	0.844	0.762
FATHMM-XF	0.850	0.794	0.794	0.966	0.911	0.794
Our 400-shot predictions						
DeepSEA	0.692	0.618	0.655	0.722	0.739	0.660
CNN+MLP (GM12878)	0.710 ± 0.028	0.635 ± 0.036	0.826 ± 0.006	0.628 ± 0.115	0.691 ± 0.046	0.755 ± 0.042
CNN+MLP (IMR90)	0.757 ± 0.024	0.670 ± 0.018	0.918 ± 0.063	0.746 ± 0.021	0.775 ± 0.056	0.723 ± 0.038
CNN+MLP (K562)	0.744 ± 0.017	0.672 ± 0.030	0.846 ± 0.056	0.654 ± 0.056	0.745 ± 0.033	0.803 ± 0.024
DanQ	0.760	0.705	0.924	0.673	0.763	0.647
CNN/RNN+GCN (DNABERT) (GM12878)	0.736 ± 0.008	0.669 ± 0.01	0.793 ± 0.091	0.739 ± 0.061	0.695 ± 0.021	0.821 ± 0.031
CNN/RNN+GCN (DNABERT) (IMR90)	0.766 ± 0.016	0.710 ± 0.028	0.895 ± 0.053	0.708 ± 0.101	0.730 ± 0.054	0.799 ± 0.042
CNN/RNN+GCN (DNABERT) (K562)	0.790 ± 0.014	0.743 ± 0.020	0.878 ± 0.047	0.724 ± 0.045	0.757 ± 0.032	0.804 ± 0.023

Table S25: Comparing our proposed methods (400-shot) and the SOTA models on the subset of test set where all models can make predictions. AUPRCs are reported.

	All (2.37%)	Intronic (1.33%)	Intergenic (6.59%)	ncRNA (0.93%)	3'utr (8.70%)	5'utr (7.99%)
Conservation-based predictor						
PhyloP 100-way	0.214	0.121	0.452	0.401	0.376	0.341
Predictors trained on large training sets (up to tens of millions)						
CADD v1.4	0.318	0.127	0.734	0.298	0.649	0.392
DANN	0.115	0.023	0.733	0.103	0.485	0.196
FATHMM-XF	0.324	0.178	0.315	0.551	0.617	0.314
Our 400-shot predictions						
DeepSEA	0.061	0.020	0.258	0.058	0.269	0.150
CNN+MLP (GM12878)	0.106 ± 0.027	0.026 ± 0.009	0.378 ± 0.144	0.061 ± 0.066	0.281 ± 0.092	0.295 ± 0.036
CNN+MLP (IMR90)	0.151 ± 0.019	0.033 ± 0.011	0.694 ± 0.045	0.119 ± 0.040	0.411 ± 0.088	0.267 ± 0.018
CNN+MLP (K562)	0.149 ± 0.012	0.035 ± 0.006	0.304 ± 0.034	0.054 ± 0.067	0.354 ± 0.067	0.431 ± 0.063
DanQ	0.135	0.037	0.635	0.041	0.392	0.223
CNN/RNN+GCN (DNABERT) (GM12878)	0.159 ± 0.037	0.051 ± 0.019	0.301 ± 0.185	0.038 ± 0.014	0.291 ± 0.025	0.460 ± 0.092
CNN/RNN+GCN (DNABERT) (IMR90)	0.162 ± 0.010	0.047 ± 0.003	0.461 ± 0.178	0.027 ± 0.016	0.331 ± 0.062	0.429 ± 0.059
CNN/RNN+GCN (DNABERT) (K562)	0.189 ± 0.025	0.068 ± 0.021	0.517 ± 0.039	0.068 ± 0.085	0.418 ± 0.091	0.417 ± 0.054

Our predictors outperformed PhyloP (in AUROC) and DANN (in AUROC and AUPRC) but were outperformed by CADD and FATHMM-XF. We also note that, compared to CADD and FATHMM-XF that used extensive features including annotations and large training data up to tens of millions of variants, our predictors were free of feature engineering and trained over only 400 variants.

We went ahead to show that our predictors, being annotation-free, more applicable, and trained on few data, can complement SOTA models and improve their performances. We first standardized the SOTA models' predictions into the interval [0, 1] where higher values mean more likely pathogenicity. Then, for each variant, we calculated the average prediction score of the SOTA and our models. We report the AUROC and the AUPRC of the pathogenic variant prediction in the following table. When our CNN/RNN+GCN with DNABERT was combined with CADD and FATHMM-XF, respectively, it reached the highest AUROC (0.86) and the highest AUPRC (0.36–0.37).

Table S26: Ensembles of SOTA and our 400-shot predictors, especially CNN/RNN+GCN w/ DNABERT, further improve SOTA predictors' performances.

	CADD v1.4 only	+ CNN/MLP	+ CNN/RNN+GCN (DNABERT)
AUROC	0.856	0.856±0.001 / 0.855±0.001 / 0.859±0.004	0.860±0.005 / 0.857±0.001 / 0.860±0.003
AUPRC	0.318	0.306±0.028 / 0.294±0.024 / 0.341±0.017	0.361±0.029 / 0.357±0.010 / 0.360±0.020

	DANN only	+ CNN/MLP	+ CNN/RNN+GCN (DNABERT)
AUROC	0.762	0.763±0.001 / 0.762±0.001 / 0.766±0.002	0.769±0.005 / 0.764±0.001 / 0.769±0.003
AUPRC	0.115	0.134±0.018 / 0.151±0.011 / 0.157±0.013	0.186±0.037 / 0.169±0.012 / 0.174±0.018

	FATHMM-XF only	+ CNN/MLP	+ CNN/RNN+GCN (DNABERT)
AUROC	0.850	0.853±0.004 / 0.858±0.005 / 0.852±0.000	0.852±0.001 / 0.853±0.001 / 0.856±0.002
AUPRC	0.324	0.309±0.022 / 0.309±0.011 / 0.340±0.012	0.368±0.032 / 0.362±0.012 / 0.357±0.017

References

[1] Yunhai Luo, Benjamin C Hitz, Idan Gabdank, Jason A Hilton, Meenakshi S Kagda, Bonita Lam, Zachary Myers, Paul Sud, Jennifer Jou, Khine Lin, et al. New developments on the encyclopedia of dna elements (encode) data portal. *Nucleic acids research*, 48(D1):D882–D889, 2020.

[2] Thomas N Kipf and Max Welling. Semi-supervised classification with graph convolutional networks. *arXiv preprint arXiv:1609.02907*, 2016.

[3] Jian Zhou and Olga G Troyanskaya. Predicting effects of noncoding variants with deep learning–based sequence model. *Nature methods*, 12(10):931–934, 2015.

[4] Daniel Quang and Xiaohui Xie. Danq: a hybrid convolutional and recurrent deep neural network for quantifying the function of dna sequences. *Nucleic acids research*, 44(11):e107–e107, 2016.

[5] Kathleen M Chen, Aaron K Wong, Olga G Troyanskaya, and Jian Zhou. A sequence-based global map of regulatory activity for deciphering human genetics. *Nature genetics*, 54(7):940–949, 2022.

[6] Jian Zhou, Chandra L Theesfeld, Kevin Yao, Kathleen M Chen, Aaron K Wong, and Olga G Troyanskaya. Deep learning sequence-based ab initio prediction of variant effects on expression and disease risk. *Nature genetics*, 50(8):1171–1179, 2018.

[7] William McLaren, Laurent Gil, Sarah E Hunt, Harpreet Singh Riat, Graham RS Ritchie, Anja Thormann, Paul Flicek, and Fiona Cunningham. The ensembl variant effect predictor. *Genome biology*, 17(1):1–14, 2016.

[8] Harry Biggs, Padmini Parthasarathy, Alexandra Gavryushkina, and Paul P Gardner. ncvardb: a manually curated database for pathogenic non-coding variants and benign controls. *Database*, 2020, 2020.

[9] Katherine S Pollard, Melissa J Hubisz, Kate R Rosenbloom, and Adam Siepel. Detection of nonneutral substitution rates on mammalian phylogenies. *Genome research*, 20(1):110–121, 2010.
